# Material properties of biomolecular condensates emerge from nanoscale dynamics

**DOI:** 10.1073/pnas.2424135122

**Published:** 2025-06-02

**Authors:** Nicola Galvanetto, Miloš T. Ivanović, Simone A. Del Grosso, Aritra Chowdhury, Andrea Sottini, Daniel Nettels, Robert B. Best, Benjamin Schuler

**Affiliations:** ^a^Department of Biochemistry, University of Zurich, Zurich 8057, Switzerland; ^b^Department of Physics, University of Zurich, Zurich 8057, Switzerland; ^c^Computational Biophysics Section, Laboratory of Chemical Physics, National Institute of Diabetes and Digestive and Kidney Diseases, National Institutes of Health, Bethesda, MD 20892-0520

**Keywords:** complex coacervates, biomolecular condensates, protein dynamics, Rouse model, intrinsically disordered proteins

## Abstract

Biomolecular condensates form by phase separation of biological polymers and have important functions in the cell—functions that are inherently linked to their physical properties at different scales. A notable aspect of such membraneless organelles is that their viscoelastic properties can vary by orders of magnitude, but it has remained unclear how these pronounced differences are rooted in the nanoscale dynamics at the molecular level. Here, we investigate a series of condensates formed by complex coacervation of highly charged disordered proteins and polypeptides that span about two orders of magnitude in bulk viscosity. We find that their viscosity is highly correlated with protein translational diffusion and nano- to microsecond chain dynamics. Remarkably, analytical relations from polymer physics can predict condensate viscosity from diffusivity and chain dynamics, and vice versa, even for more hydrophobic disordered proteins and for synthetic polyelectrolytes, indicating a mechanistic link across several decades of length- and timescales. Atomistic simulations reveal that the observed differences in friction—a key quantity underlying these relations—reflect differences in interresidue contact lifetimes as a function of arginine content and salt concentration, leading to the vastly different dynamics among condensates. The rapid exchange of interresidue contacts we observe may be a general mechanism for preventing dynamic arrest in compartments densely packed with polyelectrolytes, such as the cell nucleus.

## Significance

Cells organize many of their activities within membraneless compartments known as biomolecular condensates. Our research uncovers how the rapid, small-scale motions of disordered proteins within these condensates determine their overall physical characteristics, such as viscosity and molecular transport. Remarkably, we found accurate relationships between nanoscale protein dynamics and the mesoscale behavior of condensates formed by charged, intrinsically disordered proteins. They provide quantitative predictions connecting independently measurable quantities at different scales. This finding bridges a gap in our understanding of the multiscale organization of the cell, and opens up possibilities for understanding and quantitatively influencing the properties of these essential cellular structures based on their molecular interactions.

A substantial fraction of all cellular proteins are organized in biomolecular condensates (1) formed as a consequence of phase separation, an intriguing feature of subcellular organization (2–4). These membraneless bodies can regulate cellular homeostasis and coordinate numerous biological functions through the assembly of proteins and nucleic acids (5–7). The underlying cellular processes span a wide spectrum of time- and length-scales (8), and they are governed by the physical properties of the condensates (9) and the molecular driving forces that lead to phase separation (10–14): At the nanoscale, the rate at which biomolecules explore different conformations determines the efficiency of biochemical interactions and reactions (15–17). At the microscale, these processes and their spatial organization are controlled by the translational diffusion of biomolecules within phase-separated organelles as well as the biomolecular exchange with the environment (18, 19). At the mesoscale, material properties play a role; for example, bulk viscosity affects the fusion times of condensates (20, 21), which can influence cell fate (22, 23). This multiscale complexity poses a considerable challenge in deciphering the relationships between these dynamic processes and in establishing quantitative relations that can predict and explain the behavior of biomolecular condensates. The nanoscale dynamics of biomolecular conformations are expected to be related to translational diffusion (24) and to the emergent bulk viscosity of the percolated network they form (25). Material properties ultimately derive from the interaction strengths among the biomolecules that drive phase separation, and therefore from their specific amino acid sequences (26–33), but how molecular and mesoscale dynamics are linked quantitatively is an open question (34).

A biological example with this multiscale complexity is the cell nucleus (7, 35, 36), which is rich in highly charged biomolecules. To compensate for the high negative net charge of DNA, highly positively charged proteins, such as histones and protamines, have evolved to compact the chromosomes (37, 38). Other highly charged intrinsically disordered proteins (IDPs) are involved in remodeling chromatin and in regulating gene expression and replication. For instance, the negatively charged prothymosin α (ProTα) can sequester histone H1 and accelerate its dissociation from nucleosomes (39, 40). The two oppositely charged disordered proteins histone H1 and ProTα form viscous droplets by complex coacervation at near-physiological salt concentrations, but maintain surprisingly rapid dynamics at the molecular level (41). However, viscosities and chain dynamics are expected to depend on the amino acid composition of these biological polyelectrolytes as well as the solution conditions, especially the salt concentration. Here, we aim to identify general relations between the molecular and mesoscopic properties of biomolecular condensates across a wide range of dynamics.

We focus on complex coacervates between highly charged intrinsically disordered proteins and peptides. In the condensates they form, associative phase separation is driven by electrostatic interactions (42–45) and is thus highly sensitive to salt concentration and the type of charged residues (46, 47). We employ a combination of single-molecule techniques to investigate the conformational and translational dynamics of the polypeptides, and microrheology to assess mesoscale properties. We find that the chain dynamics of intrinsically disordered proteins within these condensates range from hundreds of nanoseconds to tens of microseconds. These reconfiguration times correlate linearly with the translational diffusion coefficients of the proteins and the bulk viscosity of the condensates. From large-scale all-atom molecular dynamics (MD) simulations, we find that low salt concentrations and especially the presence of arginine residues increase the lifetimes of interchain contacts, which slows down larger-scale condensate dynamics. Altogether, we thus demonstrate a direct relation between the nanoscopic dynamics of protein chain reconfiguration, microscale translational motion, and mesoscopic viscosity within biomolecular condensates. These relations can be rationalized within the framework of semidilute polymer solutions and generalized to predict the behavior of other condensates across scales.

## Results

### Phase Separation of Biological Polyelectrolytes.

**Fig. 1.**
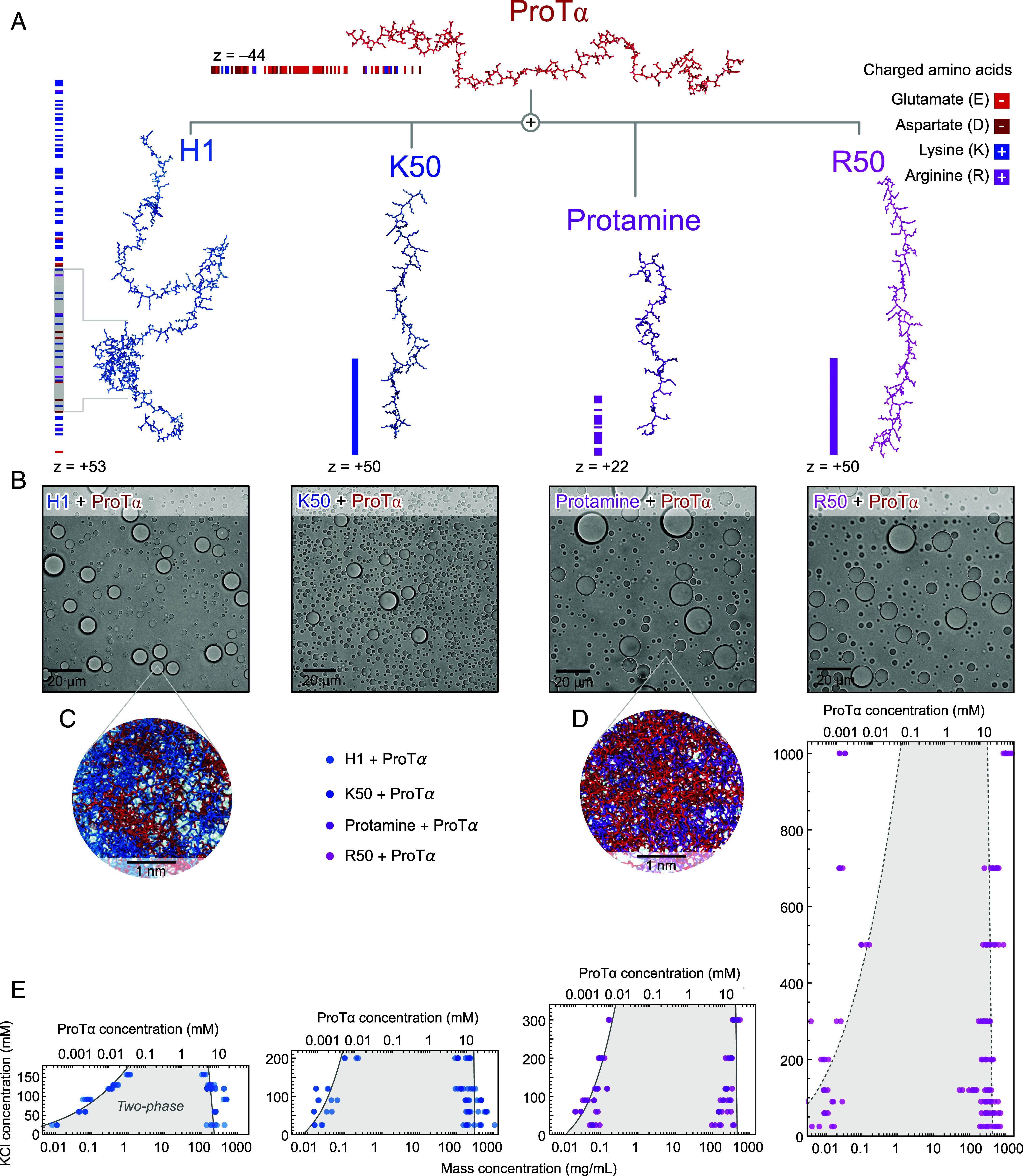
Phase separation of charged polypeptides strongly depends on their amino acid sequences. (*A*) Illustration of molecular systems used in this study with the distributions of charges along their sequences, and net charges (z): ProTα, protamine, and H1 are naturally occurring polycationic proteins; poly-L-arginine-50 (R50) and poly-L-lysine-50 (K50) are synthetic polycations (*SI Appendix*, Table S1). The gray band in the H1 sequence indicates the globular domain. (*B*) Brightfield microscopy images of phase-separated samples of ProTα mixed with a polycation (protamine, H1, R50, or K50) at charge balance in TEK buffer at 90 mM KCl (ionic strength 98 mM). (Scale bar, 20 μm.) (*C*) Illustration of the polymer networks on the nanoscale in the dense phases of H1 + ProTα and (*D*) protamine + ProTα from MD simulations. (*E*) Phase diagrams from coexistence measurements of dense and dilute phases as a function of salt concentration. The total mass concentration of both components (*Bottom* axis) is based on the measured ProTα concentrations (*Top* axis) and the charge-balanced ratio at which ProTα and the positive partner were mixed (41) (ProTα:H1 1.2:1, ProTα:K50 1.13:1, ProTα:protamine 0.5:1, ProTα:R50 1.13:1; see *SI Appendix*, Fig. S1). Phenomenological fit with a binodal curve based on Voorn–Overbeek theory (53) (solid line, dashed for ProTα:R50 where the theory fails to capture the complex interactions of arginine beyond electrostatics).

To be able to assess the influence of amino acid sequence and composition, we used the highly negatively charged disordered protein ProTα in combination with four positively charged IDPs and polypeptides with different charge densities and amino acid compositions (Fig. 1*A*): the lysine-rich histone H1 (net charge +53), the arginine-rich protamine (net charge +22), and two disordered homopolypeptides with 50 lysine (K50) or arginine (R50) residues, respectively (both net charge +50). The strong electrostatic interactions between ProTα and each of the four positively charged partners leads to associative phase separation when mixed at charge-balanced stoichiometries (*SI Appendix*, Fig. S1), as expected for oppositely charged polyelectrolytes (44, 45). Viewed under a light microscope, all phase-separated samples appear visually indistinguishable, with an aqueous dilute phase and spherical droplets of dense phase (Fig. 1 *B*–*D*). For all of them, the dense phase has a total protein mass concentration above 100 mg/mL. However, phase separation of the four samples responds differently to salt concentration: Phase separation is difficult to achieve above 200 mM KCl for the lysine-rich polypeptides, but the arginine-rich polypeptides readily phase-separate with ProTα at higher salt—for R50 even above 1 M KCl. This observation and the corresponding phase diagrams (Fig. 1*E*) highlight quantitative differences in the nature of the interactions of these two positively charged residues (47–51) related to differences in their chemical structure, charge distribution, and polarizability (52). But how do these different interactions affect the conformations and dynamics of the polypeptides that make up the condensates, as well as the corresponding mesoscopic properties?

### Condensate Dynamics across Scales.

**Fig. 2.**
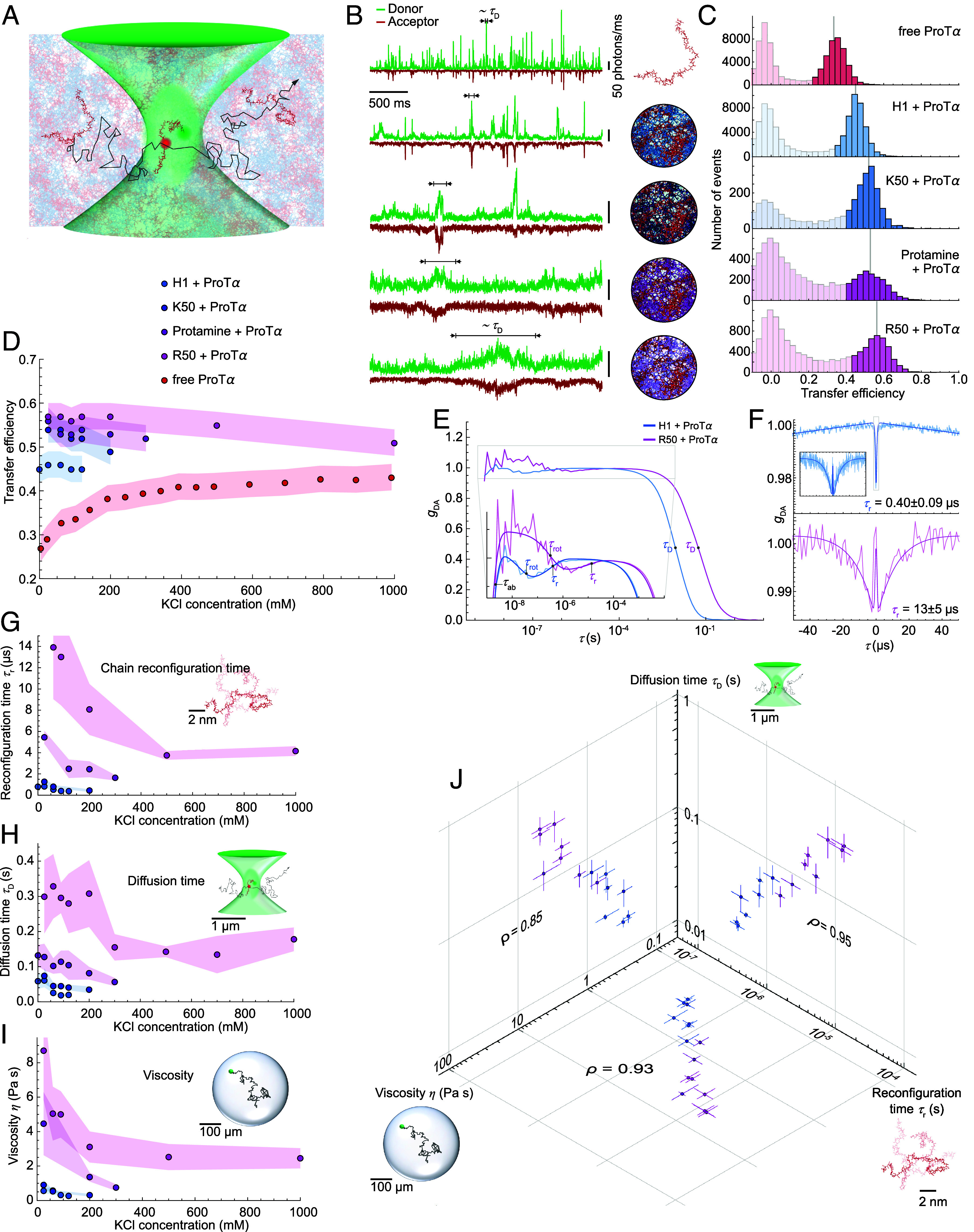
Single-molecule spectroscopy and microrheology in phase-separated droplets. (*A*) Illustration of a double-labeled ProTα molecule in the dense phase diffusing through the confocal volume. (*B*) Fluorescence time traces of (from *Top* to *Bottom*) double-labeled ProTα as a monomer free in solution, in complex coacervate droplets of ProTα + H1, ProTα + K50, ProTα + protamine, and ProTα + R50. The diffusion time, *τ*_D_, is the average time it takes a single labeled ProTα molecule to transit the confocal volume, resulting in a fluorescence burst. (*C*) Single-molecule transfer efficiency histograms of double-labeled ProTα as a monomer in solution and within droplets (ordered as in *B*) in TEK buffer at 90 mM KCl (ionic strength 98 mM). To minimize the contribution of donor-only molecules and the influence of photobleaching, fluorescence bursts corresponding to the shaded parts of the histograms were excluded from correlation analysis. (*D*) Average transfer efficiency of double-labeled ProTα as a monomer free in solution and in complex coacervate droplets as a function of salt concentration. Shaded bands represent the systematic uncertainty due to instrument calibration. (*E*) Full FCS curves with logarithmic time binning of donor-acceptor cross-correlations (g_DA_) normalized to an amplitude of 1 at 10 μs for ProTα + H1 and 100 μs for ProTα + R50, respectively, to facilitate direct comparison (*τ*_rot_, segmental rotational correlation time; *τ*_r_, chain reconfiguration time; *τ*_D_, translational diffusion time). (*F*) Representation of FCS curves with linear time binning in the range where chain dynamics dominate the signal. (*G*) ProTα reconfiguration time, *τ*_r_, in the different coacervates as a function of salt concentration obtained from the FCS fits as shown in (*E* and *F*) (*Methods*). Error bands, SD from three measurements or the error of the fit of *τ*_r_, whichever was greater (*Methods*). (*H*) Translational diffusion time through the confocal volume of double-labeled ProTα in the different coacervates as a function of salt concentration obtained from the FCS fits as shown in (*E*) (*Methods*). Error bands, SD from n ≥ 3 measurements. (*I*) Viscosity, *η*, from measurements of translational diffusion of 100- and 500-nm polystyrene beads within the different coacervates from particle tracking (*Methods*) as a function of salt concentration. Error bands, SD from n ≥ 20 tacked beads. (*J*) Correlations between the data in (*G*, *H*, and *I*) indicate a physical relation between *τ*_r_, *τ*_D_, and *η*. The slopes from linear regression of the data, plotted on a log scale, are 1.3 ± 0.2 for *τ*_D_ vs. *τ*_r_; 1.2 ± 0.2 for *η* vs. *τ*_r_; 0.8 ± 0.2 for *η* vs. *τ*_D_ (uncertainties represent 95% CI, *ρ* are the Pearson correlation coefficients).

To probe the conformations and intrachain dynamics of individual proteins within the different dense phases at the nanoscale, we used confocal single-molecule Förster resonance energy transfer (FRET) spectroscopy (54). We prepared droplets with unlabeled samples and doped them with ProTα double-labeled with Cy3B as a donor and CF660R as an acceptor fluorophore at positions 56 and 110 to monitor intramolecular distances and distance fluctuations. The doping ratio between labeled and unlabeled protein was adjusted to yield a final concentration of ~100 pM labeled ProTα within the droplets to enable FRET measurements with single-molecule resolution (Fig. 2 *A* and *B*). The resulting FRET efficiency histograms (Fig. 2*C*) show that free monomeric ProTα in dilute solution is expanded at low salt concentration, resulting in a low mean transfer efficiency, ⟨*E*⟩, due to the repulsion between the negative charges along the chain. The repulsion is screened at high salt, leading to chain compaction (55, 56) (Fig. 2*D*). The higher FRET efficiencies of ProTα inside the condensate droplets indicate chain compaction, which increases with the charge density and the arginine content of the polycationic interaction partners, reflecting stronger interactions with ProTα (Fig. 2*C*). In contrast to the free monomeric chain, ProTα within the droplets experiences a slight expansion with increasing salt concentration, as indicated by a decrease in ⟨*E*⟩ (Fig. 2*D*). Since we observe no significant correlation between protein mass concentration and chain dimensions (*SI Appendix*, Fig. S2*A*), the most likely cause of this expansion is the screening of the electrostatic attraction between oppositely charged chains by salt.

The intrinsically disordered protein ProTα samples a heterogeneous ensemble of conformations within the droplets (41). We measured the corresponding chain relaxation (25) or reconfiguration time, *τ*_r_, by monitoring the fluctuations of the acceptor-donor distance using single-molecule FRET combined with nanosecond fluorescence correlation spectroscopy (nsFCS) (57, 58) (Fig. 2 *E* and *F* and *Methods*). We find that the amino acid composition of the polycationic partner strongly influences the chain dynamics of ProTα in the dense phases. ProTα and the lysine-rich H1 form droplets in which the protein rearrangements are extremely fast, with *τ*_r_ of hundreds of nanoseconds (41), whereas in arginine-rich droplets, chain reconfiguration is slowed down by more than an order of magnitude, with *τ*_r_ exceeding 10 μs in some cases (Fig. 2 *F* and *G*). In addition to the dependence on sequence composition, *τ*_r_ in the droplets increases with decreasing salt concentration, by a factor of about 2 to 3 across the salt concentrations accessible for the different condensates (Fig. 2*G*). This observation is consistent with the hypothesis that ions screen the intermolecular interactions that slow down chain rearrangements, as reflected by the moderate chain expansion at high salt concentration (Fig. 2*D*).

From the FCS measurements (Fig. 2*E*), we can also extract the diffusion time, *τ*_D_, of the labeled protein molecules through the confocal volume (Fig. 2*H* and *Methods*). While *τ*_r_ reports on the nanoscopic dynamics within the polypeptide chain, *τ*_D_ provides information on the translational motion of the protein through the percolated network of the condensate on the micrometer length scale of the confocal volume and is inversely proportional to the diffusion coefficient. The dependence of the translational diffusion of ProTα on the sequence composition of the binding partner and the salt concentration shows remarkably similar trends as the nanoscopic chain dynamics (compare Fig. 2 *H* and *G*): ProTα diffuses more rapidly in droplets with lysine-rich than with arginine-rich interaction partners and at high than at low salt concentrations.

To characterize the mesoscopic dynamics of the condensates, we used microrheology and tracked the diffusion of fluorescent beads of 100 and 500 nm diameter inside the droplets (*SI Appendix*, Fig. S3 *A* and *B*). From the mean squared displacement, we obtained the viscosity from the Stokes–Einstein relation (*Methods*). Viscosity is a length-scale-dependent quantity in condensates (41, 59), but in this study, we focus on the bulk viscosity by using beads much larger than the correlation lengths of the protein networks (25) (*Methods*). The viscosity in the droplets is remarkably different for complex coacervates with different polycationic proteins and ranges from ~300 to ~10,000 times the viscosity of water (Fig. 2*I*). These values remain constant for days in a given sample, indicating the absence of aging effects (60) over this period.

In summary, the salt concentration and especially the amino acid sequence composition have a strong influence on the dynamic properties of the condensates across length- and timescales, from the nanoscopic chain reconfiguration time and the microscopic translational diffusion time of molecules to the viscosity at the mesoscopic scale of entire droplets (Fig. 2 *G*–*I*). The changes span nearly two orders of magnitude for each of the physical properties studied, with remarkably high correlations between them (Fig. 2*J*), suggesting an underlying causal link across scales. To identify the molecular origins of the experimentally observed behavior, we turned to large-scale MD simulations.

### Interaction Dynamics from Atomistic Simulations.

**Fig. 3.**
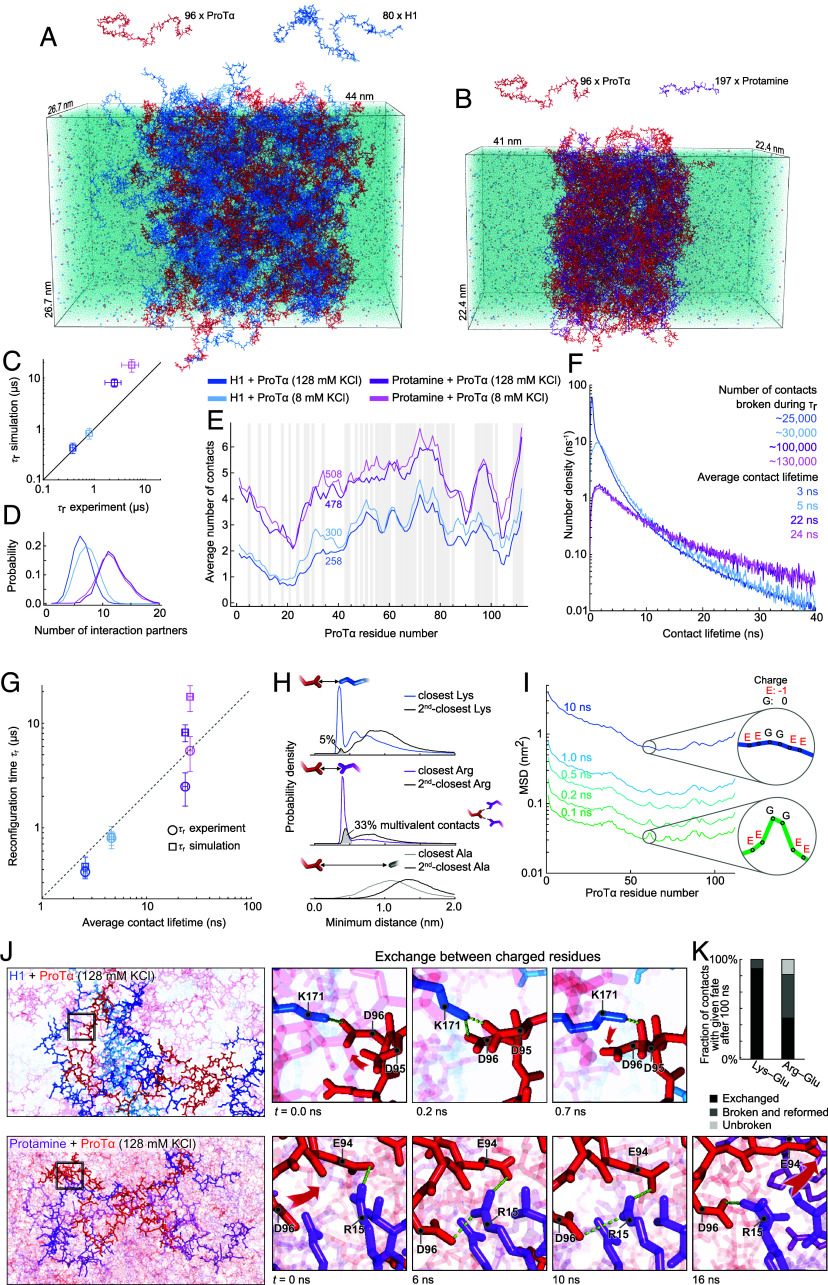
All-atom simulations of dense phases at different salt concentrations. (*A*) All-atom explicit-solvent simulations of 96 ProTα (red) and 80 H1 molecules (blue) and (*B*) of 96 ProTα (red) and 197 protamine molecules (purple) in slab geometry (41, 61), including water (transparent blue spheres), K^+^ ions (blue spheres), and Cl^−^ ions (red spheres). (*C*) Comparison between the experimental chain reconfiguration times, *τ*_r_, and the corresponding distance decorrelation times between residues 58 and 112 (corresponding to the dye positions in the experiments) from simulations (*τ*_r_ of protamine–ProTα slab at 8 mM KCl concentration is compared with the value measured at 25 mM KCl due to experimental limitations in performing stable single-molecule recordings at lower salt conditions; for uncertainties; see *Methods*). (*D*) Distribution of the number of H1 and protamine molecules simultaneously in contact with a single ProTα. (*E*) Average number of contacts (Cα atoms closer than 1 nm) made by each residue of ProTα in the four dense phases, with the average total number of contacts indicated. Gray bands indicate negatively charged residues. (*F*) Distribution of the lifetimes of contacts made by ProTα residues in the four dense phases (see *Methods* for detailed contact lifetime definition). We also report the estimated number contacts of a single ProTα chain that break during its reconfiguration time. (*G*) The correlation between the average contact lifetime of individual ProTα residues with residues in other chains (averages over all residues of all ProTα molecules in each simulation) and the chain reconfiguration time suggests a frictional effect of intermolecular contacts slowing down chain dynamics (for uncertainties, see *Methods*). (*H*) Distance distribution of the closest and the second-closest lysine (charge +1), arginine (+1), and alanine (0) to glutamate (−1) residues in ProTα chains (lysine and alanine distributions from H1–ProTα simulation, arginine distributions from protamine–ProTα simulation, both at 128 mM KCl). A sharp peak is present only in the distributions between oppositely charged residues. The shaded gray area represents the fraction of glutamate side chains involved in a multivalent close contact with two positively charged residues, which is sixfold greater for arginine than for lysine (*Methods*). (*I*) Mean-square displacement (MSD) of individual ProTα residues at increasing lag times shows that the lower friction (higher mobility) of uncharged residues resulting from weaker contacts is evident at short times, but is subsequently smoothed out at longer times when differences in friction for individual residues are averaged over longer chain segments. (*J*) Examples of exchange between lysine salt bridges in H1–ProTα (*Top*) and arginine salt bridges in protamine–ProTα dense phases (*Bottom*). Multivalent contacts (62) between negatively charged residues and arginine are more frequent and more stable than with lysine, as illustrated by representative snapshots from the simulations (Movies S1 and S2). (*K*) Lysine–glutamate contacts in the H1–ProTα dense phase exchange more frequently than arginine–glutamate contacts in the protamine–ProTα dense phase. Among the contacts that persist for over 100 ns, the majority do not remain intact continuously but break and reform between the same two residues (see *Methods* for details).

To be able to probe interactions at the atomic level and compare absolute timescales with experiment, we used all-atom MD simulations of the coacervates with explicit solvent in a recently validated (41) slab configuration (61) (*Methods*). To assess the role of lysine vs. arginine, we simulated systems consisting of 96 ProTα and 80 H1 molecules in one case, and 96 ProTα and 197 protamine molecules in the other. The two systems correspond to roughly 4 and 2.6 million atoms in the simulation box, respectively (Fig. 3 *A* and *B* and Movies S1 and S2). To study the effect of salt concentration, we performed simulations with 8 and 128 mM KCl for both systems.

The previous in-depth comparison of the simulations for ProTα and H1 with experimental observables, including protein concentrations, translational diffusion coefficients, intrachain distances, and chain dynamics, provided a validation of simulations with the force field and slab configuration employed (41). Moreover, the simulations at lower salt concentration and with protamine instead of H1 reproduce the higher protein concentrations (*SI Appendix*, Fig. S4) and the slower chain dynamics observed experimentally in droplets (Fig. 3*C* and *SI Appendix*, Fig. S5), indicating that the force field also captures the effect of salt and differences in amino acid-specific interactions (30).

On average, each ProTα molecule in the dense phase is simultaneously in contact with ~6 to 7 H1 or ~11 protamine molecules, respectively (Fig. 3*D*). Detailed information on the distribution of interactions between positively and negatively charged side chains in the resulting percolated network (63) can be obtained from contact profiles (Fig. 3*E*) and contact maps (*SI Appendix*, Fig. S6). The average number of contacts that each residue in ProTα makes with other chains reveals remarkably similar interaction patterns in the dense phases with the different interaction partners, with local maxima at clusters of negatively charged residues in ProTα (41, 64). The absolute numbers of contacts, however, differ substantially between the different dense phases: The average number of contacts that ProTα residues make with protamine is ~80% greater than with H1, and ~10% greater at 8 mM than at 128 mM salt. The origin of the pronounced difference in interaction strength between lysine- and arginine-rich sequences in the simulations is expected to lie in the characteristic multipole moments of arginine (65), its weak hydration (66), and large polarizability (52), although especially the latter can only be captured indirectly with nonpolarizable all-atom force fields (67).

The stronger interchain interactions at low salt and for arginine-rich sequences are thus likely to be at the root of the slower dynamics observed experimentally (Fig. 2). Indeed, the average lifetime of contacts in the dense-phase simulations of protamine-ProTα is about 10 times longer than for H1-ProTα (Fig. 3*F*). The duration of the contacts is in turn expected to be a determining factor for the motion of the polypeptide chain as a whole (29, 31, 68, 69). This expectation is corroborated by the correlation between contact lifetimes and chain reconfiguration times estimated from the simulations (Fig. 3*G*) (see *Methods* for details). The similarity between simulated and measured reconfiguration times (Fig. 3*C*) further suggests that the atomistic picture emerging from the MD simulations can help to explain the dynamics observed experimentally. Although complete equilibration of the protamine-ProTα simulations is challenging even with high-performance computing, the analysis is robust with respect to the quantities we report (*SI Appendix*, Fig. S4).

The simulations yield a picture in which charged residues form close contacts, as reflected by a pronounced short-range peak in the residue–residue distance distribution that is absent for uncharged residues (Fig. 3*H*). This interaction is also reflected in the diffusion profile of charged residues, which at short times show a lower mobility than their uncharged neighbors (Fig. 3*I*). However, these differences average out at longer times when the motion is dominated by larger chain segments. It is worth emphasizing that the contact lifetimes between individual charged residues are roughly two orders of magnitude shorter than the global reconfiguration times of the polypeptide chains. An important contribution to the short contact lifetimes is the rapid exchange between interacting side chains at the exceedingly high—roughly 1 M—concentrations of charged residues in the dense phases (41) (Fig. 3 *J* and *K*). Owing to the separation of timescales between contact lifetimes and the reconfiguration dynamics of entire chains, tens of thousands of residue–residue contacts are made and broken during *τ*_r_ (Fig. 3*F*). Correspondingly, the different strengths of side chain interactions made by lysine and arginine can also be considered to result in different average frictional forces acting on the chains rich in lysine and arginine, respectively (70).

### Universal Link between Nanoscale, Microscale, and Mesoscale Dynamics.

The effects of amino acid composition and salt concentration observed in the simulations, especially the correlation between contact lifetimes and reconfiguration times (Fig. 3*G*), imply a quantitative link between side chain interactions and larger-scale motion, as previously suggested based on coarse-grained simulations (29, 31, 32, 68, 69). Striking linear correlations are also observed between the experimental chain reconfiguration times, translational diffusion times, and droplet viscosities (Fig. 2*J*). Given these correlations across length- and timescales, we thus seek a physical model for condensate dynamics that allows us to predict microscale and mesoscale properties from nanoscale dynamics and vice versa.

**Fig. 4.**
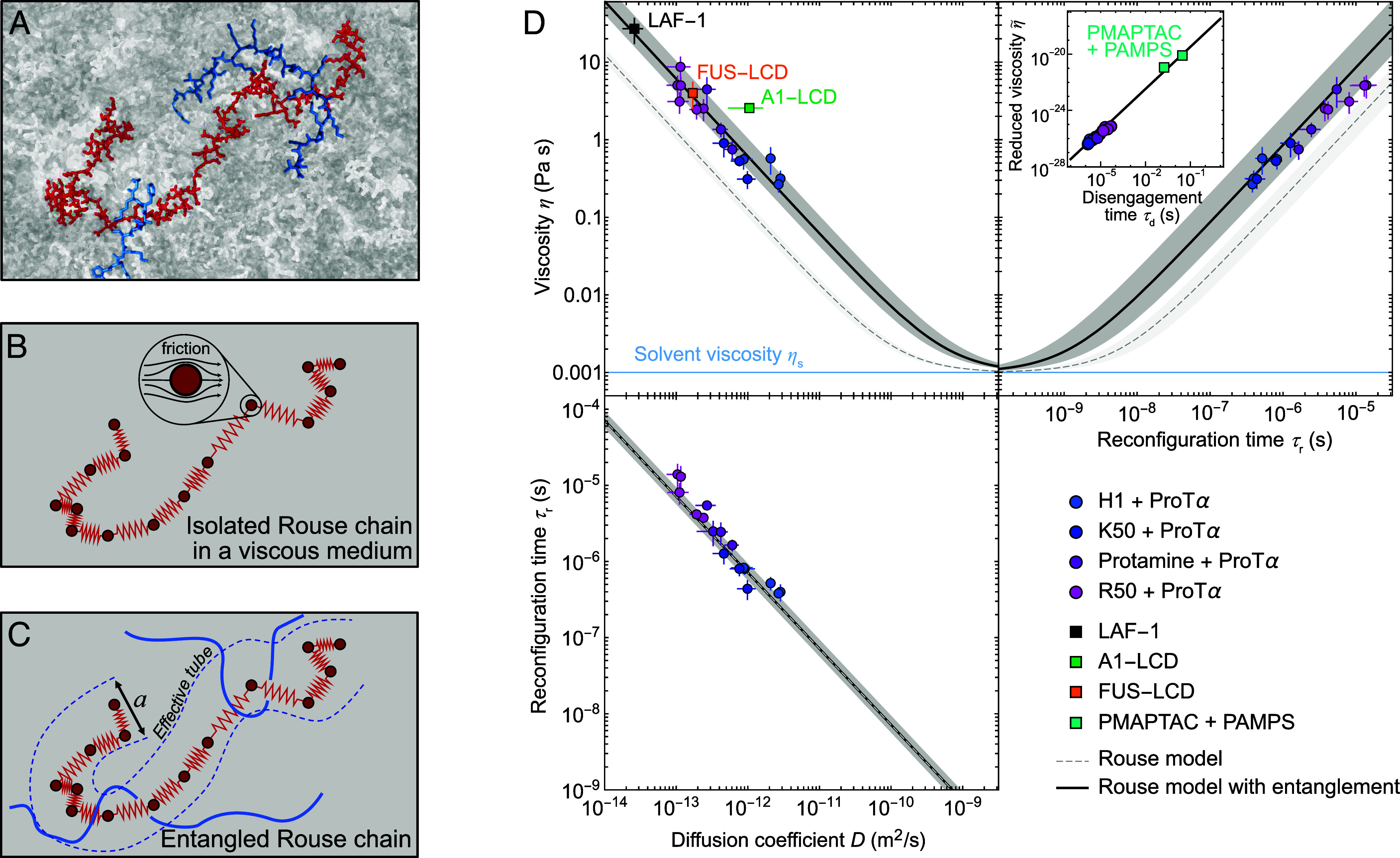
Polymer models provide a quantitative link between single-chain dynamics, translational diffusion, and bulk viscosity. (*A*) Illustration of a ProTα molecule (red) in the H1-ProTα dense phase (gray) from MD simulations, with two H1 segments entangled with ProTα shown in blue. (*B*) Schematic of the Rouse model corresponding to A, with beads (red circles) subject to Brownian motion and friction from the environment, and entropic springs connecting them. (*C*) Schematic of the Rouse model with entanglement, where the motion of a Rouse chain is constrained by a network of obstacles with a characteristic distance between them equal to *a*, the effective tube diameter (75) or entanglement spacing (76). (*D*) Comparison of the experimentally observed viscosities, diffusion coefficients, and chain reconfiguration times with the prediction of the Rouse model (dashed line) and the Rouse model with entanglement (solid line) from Eqs. **1**–**5** and a=4±2 nm (*Methods*), including the viscosities and diffusion coefficients of LAF-1 (59), A1-LCD (33, 77), and FUS-LCD (78, 79). Note that the value of *a* for these condensates may be different from the coacervates investigated here, therefore the model predictions are only indicative for these systems. For *τ*_r_(*D*), the two models overlap. The *Inset* reports the relation in Eq. **5** between the disengagement time, $\tau_{d}$ (roughly corresponding to $\tau_{r}$; see *Methods*), and the reduced viscosity, $\tilde{\eta }=\left(\eta \left(\tau_{d}\right)-\eta_{s}\right)a^{2}/c_{p}\left\langle R^{2}\right\rangle$ in Pa s m^3^, which allows us to compare our samples with PMAPTA + PAMPS (chain lengths P619 and P1188) (80). The error bands of the fits account for the differences in *a* (*SI Appendix*, Fig. S7*C*), $\left\langle R^{2}\right\rangle$, and *c*_*p*_ between the different samples. Data are presented as mean values ± SD (*Methods*).

Polymer physics presents an opportunity to conceptualize the dynamics of biomolecules in condensates across scales (Fig. 4 *A*–*C*). The residue–residue interactions observed in our simulations could be taken to suggest a model that explicitly accounts for the role of individual stickers mediating discrete binding events between the chains (71–73). However, existing quantitative models, such as the sticky Rouse model (72), typically assume bond energies greater than 10 k_B_T^*^  and correspondingly long contact lifetimes (74), and they have been developed primarily for systems with only a few stickers per chain, where chain dynamics between stickers occur faster than the contact lifetimes. These assumptions do not hold in our complex coacervates, where the nanosecond contact lifetimes suggest much lower effective bond energies, and where charged residues acting as stickers constitute a large fraction of the polymer—up to 100%. If the number of stickers approaches the total number of monomers in a chain, the sticky Rouse model converges to a standard Rouse model with increased monomer friction (74). Indeed, we are in this limit, with contact lifetimes orders of magnitude shorter than the chain reconfiguration times, so that thousands of contacts are made and broken along the chain during *τ*_r_ (Fig. 3*F*). The effect of side chain interactions can then be captured by an effective friction coefficient, rather than explicitly accounting for bond formation dynamics.

This idea is used in the Rouse model of polymer solutions (24, 25, 81) (Fig. 4*B*), which describes the dynamics of chains in terms of *N* connected segments subject to Brownian motion with a friction coefficient, ζ. The resulting relation between the translational diffusion coefficient of the entire chain, $D=\frac{k_{B}T}{N\zeta }$, and the Rouse time of the chain, *τ*_R_ (roughly the reconfiguration time, *τ*_r_; see *Methods*), is

[1] $$\tau_{R}=\frac{\left\langle R^{2}\right\rangle }{3\pi^{2}D},$$

where $\left\langle R^{2}\right\rangle$ is the mean squared end-to-end distance of the chain (*Methods*). The bulk droplet viscosity, *η*, can be expressed in terms of the friction coefficient acting on the individual chain segments, and thus in terms of the experimental observables *D* or *τ*_R_ according to

[2] $$\eta \left(D\right)=\eta_{s}+\frac{k_{B}Tc_{p}\left\langle R^{2}\right\rangle }{36}\frac{1}{D}\;\text{and}$$

[3] $$\eta \left(\tau_{R}\right)=\eta_{s}+\frac{\pi^{2}k_{B}Tc_{p}}{12}\tau_{R},$$

where $\eta_{s}$ is the solvent viscosity, and *c*_*p*_ is the protein concentration in the condensates (*Methods*). Using the experimentally measured values of *η*, *c*_*p*_, *D*, and *τ*_R_, the model correctly predicts—without any adjustable parameters—the linear dependencies observed experimentally, with absolute values within an order of magnitude of the experimental findings (Fig. 4*D*, dashed lines). The model thus explains much of the mesoscopic properties of the droplets based on the notion that a polymer chain within the droplet behaves essentially like an isolated polymer within a more viscous medium imparting friction on the chain segments. The MD simulations support this notion based on the separation of timescales between contact lifetimes and chain reconfiguration and the large number of contacts made and broken during the reconfiguration time. The proportionality between contact lifetimes and chain reconfiguration times (Fig. 3*G*) is additional evidence that friction depends on the duration of individual contacts.

However, based on the measured chain dimensions and protein concentrations, with average protein volume fractions between 17% and 31% (Fig. 1*E* and *Methods*), the dense phase is in the semidilute regime (*Methods*), where the chains partially overlap, indicating that interactions beyond purely frictional contributions may need to be taken into account. Indeed, the entanglement concentration is estimated to be in the range of the protein concentrations we observe in the dense phases (*Methods*), suggesting that we are in a regime corresponding to the onset of entanglement effects between chains (82). This conclusion is further supported by the ~4th-power dependence of viscosity on protein concentration (*SI Appendix*, Fig. S7*A*), and by the MD simulations, which indicate that every protein chain interacts with 6 to 11 other chains (Fig. 3*D*), suggesting a contribution of entanglement-like effects that restrict the reorientation of the chains within the network of other chains (81, 83). Under these conditions, the experimentally observable chain reconfiguration time, $\tau_{r}$, roughly corresponds to the disengagement time, $\tau_{d}$ (*SI Appendix*, Fig. S7*B* and *Methods*), which is identical to Eq. **1** for the Rouse model. Including entanglement in the Rouse model (Eqs. **2** and **3**) yields a correction to the expressions for viscosity by the factor $\frac{\left\langle R^{2}\right\rangle }{a^{2}}$ (76, 81), i.e.,

[4] $$\eta \left(D\right)=\eta_{s}+\frac{k_{B}Tc_{p}\left\langle R^{2}\right\rangle }{36}\frac{\left\langle R^{2}\right\rangle }{a^{2}}\frac{1}{D}\;\text{and}$$

[5] $$\eta \left(\tau_{d}\right)=\eta_{s}+\frac{\pi^{2}k_{B}Tc_{p}}{12}\frac{\left\langle R^{2}\right\rangle }{a^{2}}\tau_{d},$$

where *a* is the diameter of a tube-like region made of the surrounding polymers within which the motion of the chain is essentially confined (75, 81) (Fig. 4*C*). This effective tube diameter (75) is expected to be on the order of the mesh size (81), which we previously estimated to be ~2 to 4 nm (41), as well as the correlation length (84), which is ~1 to 5 nm (*Methods*). An analytical estimate based on protein concentration and chain dimensions (85) yields *a* = 3 ± 1 nm (*Methods*). If we treat *a* as an adjustable parameter, we obtain a value of 4 ± 2 nm from fitting Eq. **4** to the experimental data (Fig. 4*D* and *SI Appendix*, Fig. S7C), in remarkable agreement with these estimates. The value of *a* is only about half of ${\left\langle R^{2}\right\rangle }^{1/2}$, confirming that the polypeptides are only weakly entangled, as expected for such short chains (81). Nevertheless, the contribution of entanglement is essential for quantitative agreement with experiment (Fig. 4*D*). It is worth emphasizing that Eqs. **1**–**5** are relations between quantities that are measured independently, and that the predictions have no adjustable parameters except *a*, which turns out to match independent estimates for our coacervates (85).

The agreement with the Rouse model across all our coacervates prompts the question of whether its applicability is limited to complex coacervates of highly charged proteins. We thus compared with the behavior of four other phase-separated systems for which at least some of the pertinent data are available: three proteins that form biomolecular condensates and for which diffusion coefficients and bulk viscosities have been reported, LAF-1 (59), A1-LCD (33, 77),^†^ and FUS-LCD (78, 79); and a synthetic complex coacervate widely used in industrial applications, consisting of PMAPTAC (poly([3-(methacrylamido) propyl]trimethylammonium chloride)) and PAMPS (poly(2-acrylamido-2-methyl-1-propanesulfonic acid) (80), with bulk viscosities and disengagement times^‡^ roughly six orders of magnitude greater than those of the biomolecular systems. Remarkably, all of these data are in line with the behavior of the coacervates studied here (Fig. 4*D*), suggesting that the Rouse framework is more generally applicable and may provide a simple universal link between nanoscopic, microscopic, and mesoscopic properties of biomolecular condensates formed by disordered proteins and even synthetic polyelectrolytes. As a result, we can also provide order-of-magnitude estimates for the expected end-to-end reconfiguration times of the other IDPs in their dense phases: approximately 0.5 to 5 µs for A1-LCD, 3 to 30 µs for FUS-LCD, and 10 to 100 µs for LAF-1.

## Discussion

Our results demonstrate a close mechanistic link between interaction dynamics across scales: from the contact lifetimes between amino acid residues and the resulting chain dynamics at the molecular scale, to the micro- and mesoscale dynamics and viscosity of biomolecular condensates formed by charged disordered proteins. Both the amino acid composition and the salt concentration modulate the interactions and dynamics within these complex coacervates, with a particularly pronounced role for the charge density in the chains and their content of arginine, which has previously been found to be an important residue for driving biomolecular phase separation (50, 62). Key quantities reflecting the dynamics of condensates, ranging from the chain reconfiguration time and the translational diffusion of chains to the bulk viscosity of the condensates, can be linked quantitatively by the Rouse model with entanglement. Existing data on condensates whose formation is driven by hydrophobic interactions as well as complex coacervates formed by synthetic polymers suggest the existence of the same mechanistic link between scales in those systems. The success of the Rouse model for such a wide range of different biomolecules indicates that key aspects of the underlying physics of these systems are remarkably similar.

As expected from the high protein concentrations inside the condensates, and as indicated by the MD simulations of the complex coacervates investigated here as well as by previous simulations (31, 63, 86, 87), the protein chains form a highly connected network of interactions, the hallmark of viscoelastic network fluids. Despite the expected viscoelasticity of such systems, we observe the viscous component of the shear relaxation modulus to be dominant for the complex coacervates formed from highly charged disordered proteins on the accessible timescales, both in the present work and in previous results on H1 and ProTα (41). For instance, the microrheological measurements by bead tracking are well described in terms of normal Brownian diffusion down to the shortest accessible timescales in the millisecond range (*SI Appendix*, Fig. S3 *B*–*D*); for H1-ProTα, droplet relaxation upon fusion is single-exponential, with a relaxation time proportional to the radius of the final droplet, which also indicates that the viscoelasticity of the dense phase on the millisecond timescale and above is dominated by a viscous (rather than an elastic) component (20, 21). The MD simulations of H1-ProTα (41) and protamine-ProTα condensates (Fig. 3) suggest an interesting molecular mechanism contributing to the pronounced fluidity of these complex coacervates: The extreme concentration of charged side chains of >1 M in the dense phase, corresponding to an average distance between charged groups of <1 nm, facilitates the formation of transient ternary interactions between multiple charged groups (Fig. 3*H*). These interactions lead to the rapid exchange of contacts between residues (Fig. 3*J*) (41). This dynamic shuffling may be essential for many processes in the cell, e.g., to prevent dynamic arrest in compartments such as the cell’s nucleus, which is densely packed with highly charged polyelectrolytes (36, 88).

The abundant evidence for an elastic contribution to stress relaxation^§^ in other biomolecular condensates (26, 60, 89, 90) raises the question of why viscous relaxation dominates for the coacervates we studied. To address this point, we estimated the frequency dependence of the loss and storage moduli according to Rouse theory based on our experimentally defined parameters (24, 81, 84). The crossover frequency of the two moduli is predicted to be in the range of the inverse chain reconfiguration time (*SI Appendix*, Fig. S8). For the coacervates investigated here, the elastic component is thus expected to be dominant only on timescales in the microsecond range and below, which would require megahertz microrheology to be detected (91). Our observations and the relations we propose are even in agreement with measurements on synthetic complex coacervates whose reconfiguration times of ~1 s^‡^ correspond to bulk viscosities six orders of magnitude above those of our protein-based systems (80). Therefore, biomolecular condensates with pronounced elastic relaxation at lower frequencies (26, 60, 89, 90) would be expected to show correspondingly slower chain reconfiguration and much higher viscosities.

We note, however, that there are several types of biomolecular condensates that are not expected to be described by simple polymer physics, for instance condensates composed predominantly of structured molecules (92), such as folded proteins or RNA; condensates driven by interactions between folded domains mediating long-lasting crosslinks between molecules (93), whose viscoelastic moduli are thus expected to be dominated by the timescales for making and breaking crosslinks (94); or condensates that form persistent intermolecular structures or exhibit aging (33, 60). Especially the latter systems can form kinetically arrested aggregates and rigid solids (95–97), whose persistent structure and nonequilibrium properties will require residue-specific interactions and desolvation effects to be accounted for (33, 98–100). Likewise, simulations of protein–nucleic acid condensation and of systems with high charge patterning suggest the formation of local clustering (101) and caged dynamics (102), behaviors not observed in the homogeneous condensates we investigate here. In those cases, the viscoelastic behavior is expected to be more complex than described by simple polymer models.

Two important factors contribute to the success of the simple mean-field Rouse framework for predominantly viscous and isotropic condensates: One is the pronounced separation of timescales between contact lifetimes and overall chain dynamics; the resulting time averaging over vast numbers of contacts makes the concept of friction applicable on the timescale of chain reconfiguration. Another is the absence of pronounced sequence patterns in the proteins and polypeptides included in our analysis; as a result of the corresponding effective spatial averaging (Fig. 3*I*), a homopolymer model seems to provide a reasonable approximation. However, we do observe how some sequence-specific aspects govern molecular dynamics and material properties: Condensates rich in arginine exhibit dynamics roughly two orders of magnitude slower than those rich in lysine^¶^. Nevertheless, the agreement between theory and experiments across the systems suggests that the relations linking material properties and molecular dynamics serve as good first-order approximations. While these relations allow for the prediction of molecular dynamics from viscosity (and vice versa), they do not, on their own, enable the inference of either property solely from sequence composition.

Computational approaches offer the most promising path toward addressing this limitation, for instance by quantitatively relating the energetics and dynamics of molecular simulations to the viscoelasticity of condensates (31). An et al. (32) have reported that increased condensate stability correlates with low mobilities and high viscosities in coarse-grained simulations and employed active learning to identify the influence of amino acid composition and sequence patterning on the dynamic and thermodynamic properties of biomolecular condensates. We also observe correlations between some thermodynamic and dynamic properties for the condensates investigated here (*SI Appendix*, Fig. S2 *B*–*D*). It has further been suggested that the nature of the contacts formed at the residue level can be related to viscoelastic properties via the eigenvalue spectra of Rouse-Zimm matrices that account for intra- and intermolecular contacts in the Rouse model, albeit not yet in terms of absolute timescales (33, 103–105). Using experimentally validated atomistic explicit-solvent simulations, as presented here, may enable the development of predictive approaches for absolute molecular timescales, which, according to our results, can be linked quantitatively to material properties at the mesoscopic scale.

## Methods

ProTα was recombinantly expressed, purified, and labeled for single-molecule FRET experiments as described previously (41); human histone H1.0 was purchased from New England Biolabs (product code M2501S). Poly L-lysine hydrochloride (referred to as K50) and poly L-arginine hydrochloride (referred to as R50) were from Alamanda Polymers (Huntsville, AL; catalog numbers 000-KC050, 000-R050). Protamine was from Sigma-Aldrich (product number P4020). The details of single-molecule experiments, concentration, viscosity, diffusion coefficient measurements, and the experimental setup have been described before (41, 64). See *SI Appendix* for detailed descriptions of protein expression, purification, labeling, experimental procedures, and analysis, as well as a description of the theory and simulations.

## Supplementary Material

Appendix 01 (PDF)

Movie S1. **(Left)** All-atom explicit-solvent simulation of the ProTα–H1 condensate (total time 1 μs). One ProTα chain is highlighted in red (chain 60), and four interacting H1 chains are shown in different shades of blue. Other surrounding ProTα and H1 chains are shown semi-transparently in red and blue, respectively. **(Right)** All-atom explicit-solvent simulation of the ProTα–protamine condensate (total time 1 μs). One ProTα chain is highlighted in red (chain 30), and six interacting protamine chains are shown in three different shades of purple. Other surrounding ProTα and protamine chains are shown semi-transparently in red and purple, respectively. Both videos are centered on the center of mass of the highlighted ProTα chain. The video is shown at 2 ns per frame. To slightly smooth the motion, a filter with a time constant of 4 ns was applied to all frames. Protein hydrogen atoms, water molecules, and ions were omitted for clarity. (YouTube link to high-resolution version: https://www.youtube.com/watch?v=E4Idah1J3N8&ab_channel=MilosIvanovic).

Movie S2. **(Left)** All-atom explicit-solvent simulation of the ProTα–H1 condensate (total time 50 ns). One ProTα chain is highlighted in red (chain 59), and four interacting H1 chains are shown in different shades of blue. Other surrounding ProTα and H1 chains are shown semi-transparently in red and blue, respectively. **(Right)** All-atom explicit-solvent simulation of the ProTα–protamine condensate (total time 50 ns). One ProTα chain is highlighted in red (chain 30), and six interacting protamine chains are shown in three different shades of purple. Other surrounding ProTα and protamine chains are shown semi transparently in red and purple, respectively. Both videos are centered on the center of mass of the highlighted ProTα chain. The video is shown at 100 ps per frame. To slightly smooth the motion, a filter with a time constant of 200 ps was applied to all frames. Protein hydrogen atoms, water molecules, and ions were omitted for clarity. (YouTube link to high-resolution version: https://www.youtube.com/watch?v=4G9GOYp-Fmw&ab_channel=MilosIvanovic)

## Data Availability

Simulation data and code have been deposited in Zenodo (https://doi.org/10.5281/zenodo.7967716, https://doi.org/10.5281/zenodo.7963359, https://doi.org/10.5281/zenodo.15412591) (106–108). All other data are included in the manuscript and/or supporting information.

## Footnotes

^*^*k*_B_, Boltzmann constant; *T*, temperature.

^†^The diffusion coefficient was estimated from the radius of gyration, the diffusion time through the confocal volume, and the size of the confocal volume.

^‡^The disengagement time, roughly corresponding to the chain reconfiguration time (*Methods*), or reptation time (25), was inferred from the crossover frequency in rheological measurements. The agreement of all data with Eq. **5** (*Inset* in Fig. 4*D*) highlights the consistency between FRET-based nsFCS for direct measurements of chain dynamics and the indirect determination of those dynamics from the viscoelastic properties of polymer solutions.

^§^The relative magnitude of viscous and elastic contributions to a material’s mechanical response is timescale-dependent, with viscous behavior typically dominating at low frequencies, and elastic behavior at high frequencies. However, numerous reports describe biomolecular condensates exhibiting a dominant elastic contribution even at low frequencies relative to the timescales of dynamics at the molecular scale.

^¶^In contrast, our results indicate no significant difference between the contact dynamics of glutamate and aspartate (*SI Appendix*, Fig. S6).
