# Supplementary material for: Material properties of biomolecular condensates emerge from nanoscale dynamics
Source: Proc Natl Acad Sci U S A. 2025 Jun 2;122(23):e2424135122. doi: 10.1073/pnas.2424135122 (PMC12167954; doi:10.1073/pnas.2424135122)
Supplement: Supplementary file 1 — Appendix 01 (PDF) [file pnas.2424135122.sapp.pdf]

PNAS

Supporting Information for

Material properties of biomolecular condensates emerge from nanoscale dynamics

Nicola Galvanetto[1,2,*], Miloš T. Ivanović[1,*] , Simone A. Del Grosso[1], Aritra Chowdhury[1], Andrea Sottini[1], Daniel Nettels[1], Robert B. Best[3,*] and Benjamin Schuler[1,2,*]

[1]Department of Biochemistry, University of Zurich, Zurich, Switzerland
[2]Department of Physics, University of Zurich, Zurich, Switzerland
[3]Laboratory of Chemical Physics, National Institute of Diabetes and Digestive and Kidney Diseases, National Institutes of Health, Bethesda, MD, USA
* Corresponding authors: N. Galvanetto (n.galvanetto@bioc.uzh.ch), M. T. Ivanović (m.ivanovic@bioc.uzh.ch), R. B. Best (robert.best2@nih.gov), B. Schuler (schuler@bioc.uzh.ch)

This PDF file includes:

Supporting Methods
Figures S1 to S9
Table S1
Legends for Movies S1 to S2
SI References

Other supporting materials for this manuscript include the following:

Video S1 to S2

Supporting Information Text

Materials and Methods

Sample preparation and labeling

We used recombinant human histone H1.0 (H1; New England Biolabs, product code M2501S). Poly L-lysine hydrochloride (referred to as K50, MW = 8200 Da, degree of polymerization 45–55) and poly L-arginine hydrochloride (referred to as R50, MW = 9600 Da, degree of polymerization 45–55) were from Alamanda Polymers (Huntsville, AL, USA; catalog numbers 000-KC050, 000-R050). Protamine was from Sigma-Aldrich (product number P4020). Labeled and non-labeled ProTα were prepared as described previously (1). Labeling was achieved by introducing cysteine residues at positions 56 and 110 for attaching fluorophores (detailed protein sequences in Extended Data Table 1). Prior to labeling, the protein was reduced in phosphate-buffered saline (PBS), pH 7, containing 4 M guanidinium hydrochloride (GdmHCl) and 0.2 mM EDTA, using 10 mM Tris(2-carboxyethyl) phosphine hydrochloride (TCEP) for 60 minutes, followed by multiple (5x) buffer exchange steps to a similar PBS solution without TCEP (pH 7, 4 M GdmCl and 0.2 mM EDTA) using centrifugal filters with a 3-kDa molecular mass cutoff (Sigma-Aldrich). Labeling was achieved with Cy3B maleimide (Cytiva) and CF660R maleimide (Sigma-Aldrich) at a protein: dye ratio of 1:6:6, incubated for an hour at room temperature and overnight at 277 K. Excess dye was reacted using 10 mM dithiothreitol for ten minutes and removed by centrifugal filtration. The labeled protein was subsequently purified by reversed-phase high pressure liquid chromatography using a Reprosil Gold C18 column (Dr. Maisch, Germany), without separating the labeling permutants. The correct mass of labeled protein was confirmed by electrospray ionization mass spectrometry.

Turbidity measurements

To assess the extent of phase separation, the relative turbidity was quantified by the attenuation of light at 350 nm with a NanoDrop 2000 UV-Vis spectrophotometer (Thermo Scientific). The positively charged polypeptides were added to a fixed volume of a ProTα solution to achieve a final concentration of 10 µM ProTα and investigate a wide range of stoichiometric ratios. These experiments were carried out in TEK buffer, composed of 10 mM Tris-HCl, 0.1 mM EDTA (pH 7.4), with ionic strength adjusted using KCl. Samples were rapidly mixed via pipetting for approximately 10 seconds before measurements. Each sample was measured four times, and the results were averaged. Both protein stock solutions were diluted in identical buffers before the mixing process.

Single-molecule fluorescence spectroscopy

We performed confocal single-molecule analysis, concentration determination, and fluorescence correlation spectroscopy at 295 K with a MicroTime 200 (PicoQuant), equipped with a UPlanApo 60×/1.20-W objective (Olympus), mounted on a piezo stage (P-733.2 and PIFOC from Physik Instrumente GmbH), using a 532-nm continuous-wave laser (LaserBoxx LBX-532-50-COL-PP; Oxxius), and a 635-nm diode laser (LDH-D-C-635M; PicoQuant). Fluorescence photons, which were separated from scattered laser light using a triple-band mirror (zt405/530/630rpc from Chroma), were initially separated into two channels by either a polarizing or a 50/50 beam splitter, and then into four detection channels with dichroic mirrors to separate donor and acceptor emission (T635LPXR from Chroma). Donor emission was further filtered with an ET585/65m band-pass (Chroma), and acceptor emission with an LP647RU long-pass filter (Chroma), before being detected by SPCM-AQRH-14-TR single-photon avalanche diodes (PerkinElmer). SymPhoTime 64 version 2.4 (PicoQuant) was used for data acquisition.

In single-molecule measurements, ProTα, labeled with Cy3B and CF660R, was excited by the 532-nm laser. Experiments were conducted in TEK buffer, including different concentrations of KCl. Plastic sample chambers (µ-Slide, ibidi) were used to mitigate surface adhesion of the positively charged polypeptides to glass. For measurements of dilute-phase samples, the power of the 532-nm laser was set to 100 µW (measured at the back aperture of the microscope objective); the confocal volume was positioned 30 µm deep into the sample chamber; and concentrations of labeled protein between 50 and 100 pM were used. For single-molecule measurements in the

dense phase, the average power at the back aperture was between 3 and 30 μW for continuous-wave excitation, depending on the background level; the confocal volume was placed at the center of the spherical droplets, whose radii varied between 4 and 30 μm. Unlabeled proteins (12 μM ProTα and a concentration of the positively charged polypeptides to ensure charge balance) were mixed with 5 to 10 pM of labeled ProTα. Photon bursts, occurring as proteins traverse the confocal volume, were isolated from background-corrected fluorescence trajectories, binned at 4 ms, with a photon count threshold of 285 per burst. In dilute-phase measurements, bursts were defined as sequences of at least 285 consecutive photons with interphoton times below 40 μs.

Ratiometric transfer efficiencies were obtained from $E = N_A/(N_A + N_D)$, where N_A and N_D are the numbers of donor and acceptor photons, respectively, in each photon burst, corrected for background, channel crosstalk, acceptor direct excitation, differences in quantum yields of the dyes, and detection efficiencies (2, 3). Mean transfer efficiencies, $\langle E \rangle$, were determined from fits with Gaussian peak functions to the transfer efficiency histograms. To infer dye-to-dye distance distributions, *P*(*r*), from $\langle E \rangle$, we use the relation (4)

$$\langle E \rangle = \langle \varepsilon \rangle \equiv \int_0^\infty \varepsilon(r) P(r) dr, \qquad \text{Eq. 6}$$

where

$$\varepsilon(r) = R_0^6/(R_0^6 + r^6). \qquad \text{Eq. 7}$$

The Förster radius, R_0, (5) of 6.0 nm for Cy3B/CF660R in water (6) was corrected for the refractive index, *n*, in the droplets according to the published dependence of *n* on the protein concentration (7), which is linear up to a mass fraction of at least 50 % (8) and only marginally dependent on the type of protein (7). At the dense-phase protein concentrations observed here, *n* is greater than the value in water by 3%–6%, resulting in a slightly smaller R_0 inside the droplets (5.8–5.9 nm). Systematic uncertainties in transfer efficiencies due to instrument calibration and uncertainty in R_0 are estimated to be roughly ±0.03, in line with the range previously reported (2). The precision for measurements on the same instrument is higher, typically <0.01 (6). *P*(*r*) was estimated using the length scaling exponent ν by applying an empirically modified self-avoiding-walk polymer (SAW-ν) model (9, 10). To estimate the mean square end-to-end distance of the complete ProTα chain, we used ν and the total number of amino acids, N_{tot} = 110. The impact of fluorophore labeling on ProTα-H1 interaction was minimal, as evidenced by previous studies (1, 11). Given the fraction of $<10^{-6}$ of labeled protein in the dense phases, effects of fluorophore labeling on dense-phase behavior were considered negligible. Analysis of fluorescence data was performed with the software package Fretica (https://github.com/SchulerLab) run with Mathematica 12.3 (Wolfram Research).

Measurements of protein concentrations and diffusion coefficients in the dilute and dense phases
We utilized fluorescence correlation spectroscopy (FCS) and quantitative fluorescence intensity analyses using a MicroTime 200 (PicoQuant) to assess the concentrations of ProTα molecules doubly labeled with Cy3B and CF660R, in the dense and dilute phases (12, 13). A mixture of unlabeled proteins (12 μM ProTα and a concentration of the respective positively charged polypeptides to ensure charge balance, except for the highest salt concentrations in H1–ProTα samples where we used up to 70 μM ProTα), doped with a small concentration (~10 pM to 10 nM) of labeled ProTα in TEK buffer including the specified concentrations of KCl was allowed to phase-separate at 295 K. To analyze the dilute phase, the phase-separated mixture was centrifuged at 295 K for 30 minutes at 25,000 g, leading to the formation of a single large droplet of dense phase. The supernatant was then aspirated and placed into a sample chamber (μ-Slide, ibidi). For measurements in the dense phase, the phase-separated mixture was directly transferred to the sample chamber, and droplets were allowed to settle on the bottom surface of the sample chamber by gravity; the boundaries of individual droplets were identified via 3D confocal imaging, and FCS and intensity measurements were performed by focusing inside the droplets.

To excite CF660R, we employed the 635-nm continuous-wave laser at 5 μW (measured at the back aperture of the objective). The emitted fluorescence photons were then separated using a polarizing beam splitter and subsequently detected by two detectors. The collected correlation data were analyzed employing a model that assumes a 3D Gaussian-shaped confocal volume:

$$G(\tau) = G_0 \left[\left(1 + \frac{\tau}{\tau_D}\right) \sqrt{1 + s^{-2} \frac{\tau}{\tau_D}} \right]^{-1}, \quad \text{Eq. 8}$$

where τ is the lag time, G_0 is the amplitude, τ_D is the translational diffusion time, and s is the ratio of the axial to lateral radii of the confocal volume. The calibration curve was generated from the analysis of samples with known concentrations (0.3, 1, 3 10, 30, and 100 nM) of labeled ProTα in TEK buffer including 120 mM KCl.

Concentrations were estimated from the average number of labeled proteins in the confocal volume, $N_p = \left(1 - \frac{n_b}{n_f}\right)^2 / G_0$, as previously described (13), where n_b is the background count rate estimated from samples without labeled protein, and n_f is the average count rate of the measurement with labeled ProTα. As an alternative method for estimating concentrations, the fluorescence intensity after background subtraction was used based on a corresponding calibration curve. Total ProTα concentrations were obtained by dividing the concentrations of labeled ProTα by the doping ratio, which was set to ensure that fluorescence intensities fell within the range of linear response of detection. This approach requires higher doping ratios for measurements in the dilute phase than for those in the dense phase. For each set of experimental conditions, a minimum of two concentration estimates were made, one using FCS and one using intensity detection. These assessments were repeated a minimum of two times to increase reliability.

Diffusion coefficients were calculated from translational diffusion times, τ_D, using a calibration curve

$$D = \frac{\Lambda}{\tau_D}, \quad \text{Eq. 9}$$

where Λ was obtained from a calibration with samples of known diffusion coefficient in water. The calibration was cross-validated in ProTα-H1 droplets with two-focus FCS (14) to account for effects of refractive index differences between dilute and dense phase on the observed translational diffusion coefficients (12).

Since maximum dense phase formation occurs at a mixing ratio close to charge balance (Figure S1), all experiments were performed by mixing ProTα and the positively charged polypeptides at this ratio. Since reproducible droplet formation becomes difficult to maintain and exceedingly sample consuming at salt concentrations near the critical point, a compromise between experimental feasibility and accessible salt concentrations was made.

Microrheology

We mixed ProTα and the positively charged polypeptides, both unlabeled, under phase separating conditions at charge balance with an aliquot of fluorescent microspheres (100 nm and 500 nm diameter, Fluoro-Max, Thermo Fisher Scientific). After centrifugation, we collected a single large droplet (diameter ≥100 μm) for observation. The motion of the beads within the droplet was tracked at 295 K using an Olympus IXplore SpinSR10 microscope equipped with a 100×/1.46 NA plan-apochromat oil immersion objective, for 300 seconds with 50 ms exposure time per image and and acquisition rate of 5 Hz. Bead trajectories were obtained using the TrackMate plugin of Image J (15) and further analyzed with MATLAB 2016b (MathWorks). We calculated the mean square displacements (MSD) in the image plane, averaging across 20 trajectories, to obtain the diffusion coefficient, D, from $\langle MSD(t) \rangle = 4Dt$, where t is the time after the start of observation.

The MSD analysis demonstrated uniform viscous properties within the droplets, as evidenced by the Brownian diffusion, the consistency between different beads probed in the droplet volume, and the uniform fluorescence intensity observed in microscopy images. We did not observe

aging effects on the timescale up to days. We estimated the viscosity, η, using the Stokes–Einstein equation assuming freely diffusing Brownian motion of particles with hydrodynamic radius R_h:

$$\eta = \frac{k_B T}{6\pi D R_h}. \quad \text{Eq. 10}$$

In this study, we were interested in the bulk viscosity of the medium that can be measured by probes that are much larger than the correlation length of the polymer network (16, 17). At short times, some MSD curves apparently deviate from the linear Brownian behavior. This effect is due to uncertainties in position determination owing to out-of-focus beads (Figure S3C, D), rather than due to possible elastic properties of the dense phase, which cannot be resolved within the time scales studied here (18) (Figure S8).

To increase the time resolution of tracking, we also tested K50-ProTα droplets in an optical tweezers instrument (C-Trap, LUMICKS, Amsterdam). K50-ProTα was the sample least prone to photodamage by the IR laser. A condensate-forming sample (3 µl) mixed with 1-µm polystyrene beads was placed on a polymer coverslip (ibidi GmbH, Germany) at the center of an enclosure formed by double-sided tape. Another polymer coverslip was placed on top of the sample, sandwiching and sealing it. The condensate sample was left to equilibrate for 30 min and then placed on the sample stage of the optical tweezers instrument equipped with a 60× water immersion objective and a bright-field camera. We trapped isolated beads within large droplets (diameter > 50 µm) with minimal laser power to prevent photodamage and the formation of optically visible bubbles at the bead surface. Bead motion was recorded with the camera at acquisition rate >300 Hz and tracked with Blulake (LUMICKS, Amsterdam). MSDs were calculated in Mathematica (Wolfram Research).

Correlation length, overlap concentration, entanglement concentration, and effective tube diameter

The correlation length in the dense phase was estimated from $\xi \approx R_g \left(\frac{c_p}{c^*}\right)^{-\frac{\nu}{3\nu-1}}$ (19), where c_p represents the total protein concentration (concentration of ProTα plus the concentration of the positively charged partner); c^* is the overlap concentration, defined as $c^* = 1/V$ with V approximating the volume occupied by a polymer chain ($V \approx 4/3\,\pi R_g^3$); R_g is the radius of gyration ($R_g \approx \sqrt{\langle R^2 \rangle}/\sqrt{6}$, where $\sqrt{\langle R^2 \rangle}$ is the mean square end-to-end distance of the polymer); and ν is the scaling exponent. The condition $c_p \gtrsim c^*$ corresponds to the transition from the dilute to the semidilute regime, marking the beginning of chain interpenetration (19, 20). The concentrations corresponding to c^* in our samples range from 7 mM to 10 mM, while the protein concentrations corresponding to c_p in the four samples range from 14 mM to 42 mM (concentration averages at the different protein compositions and salt concentrations). The correlation lengths, ξ, are between 1 nm and 5 nm; this range was estimated using the average values of R_g and c_p for the four protein combinations at the different salt concentrations; values of ν ranging from 0.5 to 0.6; and values of c^* based on the lower and upper bounds of both R_g and the theoretical limits for the hydrodynamic radius ($R_g/1.5 < R_h < R_g/0.77$) (19, 21) as chain dimensions. Obtaining a precise measured value of ν is challenging; from internal length scaling of the disordered proteins in the MD simulations (21), we estimate values of ν ranging from 0.53 to 0.63 for the different coacervates and salt concentrations. Since $c_p > c^*$ for all conditions used, the correlation lengths are thus smaller than the chain dimensions, so we expect excluded volume interactions to be screened by the overlapping chains on the length scales we are probing experimentally (19). Similarly, hydrodynamic interactions are expected to be screened under these conditions, suggesting that Rouse dynamics apply on the length scales of entire chains (19, 20). For consistency and simplicity, we thus use the corresponding value of ν = ½ throughout.

The entanglement concentration, c_e, delineates the transition from a non-entangled polymer solution to an entangled one when $c_p \gtrsim c_e$. This condition is met approximately if the protein concentration is high enough that there are at least two chains in the volume V pervaded by a single chain (see eq. 10 in ref. (22)), yielding $c_e \approx 2c^*$. As $c_p \approx c_e$ in our systems, only mild entanglement effects are expected. The effective tube diameter, a, was estimated from the experimental protein

concentration, c_p, and chain dimensions, $\langle R^2 \rangle$, following ref. (23): $a = l_k \sqrt{\alpha \left(\frac{l_k}{\rho_m {l_0}^3 p}\right)^{-2} + \beta \left(\frac{l_k}{\rho_m {l_0}^3 p}\right)^{-1} + \gamma \left(\frac{l_k}{\rho_m {l_0}^3 p}\right)^{-1/3}}$, where $l_0 = 0.4\,\text{nm}$ is the backbone bond length, $l_K = \frac{\langle R^2 \rangle}{L_c} \approx 1.8\,\text{nm}$ is the Kuhn length, L_c is the contour length of ProTα, $p = (c_p \langle R^2 \rangle)^{-1}$ is the packing length, $\rho_m \approx c_p L_c / l_0$ is the monomer number density, and α, β, and γ are obtained from the table in ref. (23). Considering the different protein concentrations and chain sizes in our complex coacervates, and the two different sets of values for α, β, and γ provided (23), we obtain $a = 3 \pm 1\,\text{nm}$.

Total volume fractions of protein, ϕ, were estimated from the measured ProTα concentrations, $c_{\text{ProT}\alpha}$ (Figure 1E), as $\phi = (M_{ProT\alpha}\, c_{ProT\alpha} \bar{v}_{ProT\alpha} + X\, M_{partner}\, c_{ProT\alpha}\, \bar{v}_{partner})$, where M is the molar mass, $\bar{v}$ is the partial specific volume, and X is the mixing ratio between the positively charged partner and ProTα to reach charge balance. We note that since the samples examined here are complex coacervates with components of similar but not identical lengths, the values must be considered approximate.

Nanosecond fluorescence correlation spectroscopy (nsFCS)

Samples for *ns*FCS measurements were prepared as outlined in the section *Single-molecule measurements*. To avoid the reduction in signal due to photobleaching of slowly diffusing molecules in the dense phase, we moved the confocal volume at constant speed (3 μm/s) following a serpentine trajectory in a horizontal plane within the droplet (12). Excitation with continuous-wave laser light of 532 nm was conducted at 3 or 30 μW (measured at the back aperture of the objective). For subpopulation-specific correlation analysis of the FRET-active species, we used photons from bursts with $\langle E \rangle > 0.15$. Autocorrelation curves of acceptor (A) and donor (D) detection channels, and cross-correlation between *A* and *D* were analyzed as described previously (12, 24). In Figure 2E we show cross correlation curves with logarithmically spaced lag times ranging from nanoseconds to milliseconds. The function used for fitting the correlations between detection channels $i, j = A, D$ was

$$G_{ij}(\tau) = G_{0,ij}\, \frac{(1 - c_{ab}^{ij}\, e^{-|\tau|/\tau_{ab}^{ij}})(1 + c_{cd}^{ij}\, e^{-|\tau|/\tau_{cd}})(1 + c_{rot}^{ij}\, e^{-|\tau|/\tau_{rot}})}{\left(1 + \frac{|\tau|}{\tau_D}\right)\left(1 + s^{-2}\frac{|\tau|}{\tau_D}\right)^{1/2}}. \quad \text{Eq. 11}$$

The three terms in the numerator with amplitudes c_{ab}^{ij}, c_{cd}^{ij}, c_{rot}^{ij}, and correlation times τ_{ab}^{ij}, τ_{cd}, τ_{rot} describe photon antibunching, conformational dynamics, and dye rotation, respectively. τ_{D} and *s* are defined as in Eq. 8. Conformational dynamics result in a characteristic pattern with a positive amplitude in the autocorrelations ($c_{cd}^{DD} > 0$ and $c_{cd}^{AA} > 0$) and a negative amplitude in the cross-correlation ($c_{cd}^{AD} < 0$), but with a common correlation time, τ_{cd}. The three correlation curves $G_{DD}(\tau)$, $G_{AA}(\tau)$, and $G_{AD}(\tau)$ were fitted globally with τ_{cd} and τ_{rot} as shared fit parameters. The relaxation time τ_{cd} was converted into the chain reconfiguration time $\tau_r = \int_0^\infty \frac{\langle r(0) r(\tau) \rangle}{\langle r^2 \rangle} d\tau$, according to the procedure previously established (25). This conversion is based on the assumption that dynamics of the inter-dye distance r can be represented as diffusive motion in a potential of mean force obtained from the distance distribution $P(r)$ by Boltzmann inversion (25, 26).

The experimental uncertainties of τ_{r} reported here are either standard deviations calculated from three measurements or the error of the fit of τ_{cd} (from which τ_{r} is derived), whichever was greater. We estimate the error of the fit from the variability resulting from fits using different lag-time intervals of the FCS data: We report as uncertainties the range of reconfiguration times obtained by using values from 0.8 ns to 8 ns as lower bounds, and from 1 ms to 6 ms as a upper bounds of the fitting window. The rotational correlation time, τ_{rot}, is approximately one order of magnitude smaller than the chain reconfiguration time, τ_{r}, and is caused by dye rotation (12).

Polymer models

We compared the experimental results of ProTα reconfiguration times, τ_r, ProTα translational diffusion coefficients, D, in condensates, and bulk viscosities η of the condensates, with corresponding values derived from three models commonly used for polymer solutions (Figure S7B): i) the Rouse model (most commonly used for concentrated solutions and melts) (27), ii) the Zimm model (most commonly used for dilute solutions) (28), and iii) the Rouse model with entanglement (29–31). Since the concentrations and chain dimensions in the dense phases are comparable in all systems studied, we tested the models using average values of these quantities rather than reduced quantities, which would not have allowed a comparison of different models with different dependencies on protein concentrations and chain dimensions for all samples at the same time. The upper and lower limits of the error bands in Figure 4D correspond to models calculated for the minimum and maximum values of the chain dimensions and protein concentrations in the set of experimentally observed values.

i) The Rouse model describes the dynamics of a chain of *N* beads connected by harmonic springs with root mean square length *b*, and subjected to Brownian motion with friction coefficient ζ. The friction coefficient of the whole chain is approximated as $N\zeta$. The translational diffusion coefficient of the center of mass of the entire chain, *D*, is given by the Einstein relation,

$$D = \frac{k_B T}{N\zeta}. \qquad \text{Eq. 12}$$

The longest relaxation time of the Rouse chain is (see ref. (31), eq. 4.37)

$$\tau_\mathrm{R} = \frac{\zeta N^2 b^2}{3\pi^2 k_B T} = \frac{\langle R^2 \rangle}{3\pi^2 D}\ . \qquad \text{Eq. 13}$$

For the second expression, we used $\langle R^2 \rangle = Nb^2$ and Eq. 12. τ_R is related to the vector reconfiguration time τ_{ij} of the experimentally probed segment between residues $i = 56$ and $j = 110$ of ProTα. This time is defined as a vector correlation time, $\tau_{ij} = \int_0^\infty \langle \boldsymbol{r}_{ij}(0) \cdot \boldsymbol{r}_{ij}(t) \rangle / \langle \boldsymbol{r}_{ij}^2 \rangle dt$, where $\boldsymbol{r}_{ij}$ is the vector between the positions of the two residues. Makarov found the useful relation (32)

$$\tau_{ij}/\tau_\mathrm{R} = \frac{\pi^2}{24} |\mu - \lambda| \{-(4 + 7\mu^2 - 12\lambda + 7\lambda^2 - 12\mu + 10\mu\lambda) + 4 - 4|\mu - \lambda| + (\mu - \lambda)^2\}, \qquad \text{Eq. 14}$$

where, in our case, $\mu = \frac{i}{N_\mathrm{ProT\alpha}}$ and $\lambda = \frac{j}{N_\mathrm{ProT\alpha}}$; with $N_\mathrm{ProT\alpha} = 112$, we obtain $\frac{\tau_{ij}}{\tau_\mathrm{R}} = 0.54$. Note that τ_{ij} is not identical to the reconfiguration time, τ_r, because the latter is defined as a scalar distance correlation time, $\tau_r = \int_0^\infty \langle r_{ij}(0) \cdot r_{ij}(t) \rangle / \langle r_{ij}^2 \rangle dt$, where $r_{ij}(t) = |\boldsymbol{r}_{ij}(t)|$ is the length of the vector. We are not aware of an analytical relation between τ_r and τ_{ij}. To assess the relation between the two times, we thus simulated the dynamics of Rouse chains, each for five million time steps, with different numbers of segments, N, ranging from 10 to 40, and with different ratios between the size of the time steps and the friction coefficients (ranging from 0.4 to 4) and found that the distance decorrelation time is smaller than the vector decorrelation time by a factor $\Xi = \frac{\tau_\mathrm{r}}{\tau_\mathrm{ij}} = 0.50 \pm 0.05$ (error is s.d. from 24 different simulations). It follows that the expected relation between the measured reconfiguration time, τ_r, and the Rouse time, τ_R, is in our case:

$$\tau_r \approx \Xi \cdot 0.54 \cdot \tau_\mathrm{R} = 0.27 \cdot \tau_\mathrm{R}. \qquad \text{Eq. 15}$$

The bulk viscosity of a polymer solution (27, 31) is given by (see eq. 32 in ref. (27) and eq. 7.33 in ref. (31)):

$$\eta = \frac{c\,\zeta}{36} N b^2 + \eta_s = \frac{k_B T c_p \langle R^2 \rangle}{36\,D} + \eta_s \qquad \text{Eq. 16}$$

where $c_p = c/N$ is the concentration of protein molecules in the condensates (number of protein molecules per volume), c is the concentration of chain segments (number of segments per volume) in the condensates, and η_s is the bulk solvent viscosity (1 mPa s in our samples). The relation between reconfiguration time and viscosity is given by combining Eq. 13, Eq. 15, and Eq. 16. The error bands in Figure 4D represent the results of the models calculated from the range of experimental chain dimensions, protein concentrations, and Ξ.

ii) The Zimm model extends the Rouse model by including hydrodynamic interactions. It recognizes that the motion of one part of the polymer chain affects the surrounding solvent, which in turn affects the motion of other parts of the chain; the Zimm model is thus considered appropriate for polymers in dilute solution. Based on the arguments presented above, we expect our coacervates to be in the Rouse regime, but since correlation lengths and chain dimensions are in a similar range, we present the analysis based on the Zimm model for direct comparison. The Zimm model relates the center-of-mass diffusion coefficient, *D*, and the chain reconfiguration time, τ_Z, to the solvent viscosity (see eq. 4.61 and 4.63 in ref. (31)), but if the viscosity in the polymer network is length scale-dependent (12), the relations can be inverted to obtain an effective solvent viscosity, η_Z, at the length scale of the chain relevant for polymer dynamics:

$$\eta_Z = \frac{8}{3\sqrt{6\pi^3}} \frac{k_B T}{\sqrt{\langle R^2 \rangle}\, D}\ , \qquad \text{Eq. 17}$$

which is equivalent to the Stokes-Einstein equation (Eq. 10) with $R_h = \frac{3\sqrt{\pi}}{8\sqrt{6}} \sqrt{\langle R^2 \rangle} \approx \frac{2}{3}\, R_g$,

$$\eta_Z = \frac{\sqrt{3\pi}\, k_B T\ \tau_1}{\sqrt{\langle R^2 \rangle}^3} \qquad \text{Eq. 18}$$

and $\tau_Z = \frac{8}{9\pi^2\sqrt{2}} \frac{\langle R^2 \rangle}{D}$. Eq. 19

However, in contrast to the Rouse model with entanglement (see iii), Zimm theory can only describe part of the relations we observe experimentally (Figure S7B).

iii) The Rouse model with entanglement (29–31) considers a Rouse chain diffusing in a network of other chains resulting in obstacles effectively forming tubes of diameter *a*, within which the chain can diffuse, where a can also be considered the 'entanglement spacing' (33) (Figure 4C). In this picture, the center-of-mass diffusion coefficient of the chains, *D*, depends both on the friction coefficient, ζ, acting on individual beads, and on the ratio between the tube diameter and the chain dimensions (see eq. 6.40 in ref. (31)),

$$D = \frac{k_B T}{3N\zeta} \frac{a^2}{\langle R^2 \rangle}\ . \qquad \text{Eq. 20}$$

The viscosity of the polymer solution can thus be expressed in terms of *D* (see eq. 7.47, 7.43, 6.19 and 6.40 in ref. (31)) as

$$\eta = \frac{k_B T c_p \langle R^2 \rangle}{36\,D} \frac{\langle R^2 \rangle}{a^2} + \eta_s\ , \qquad \text{Eq. 21}$$

and can be used to calculate the effective tube diameter for all samples (Figure S7C), resulting in a = 4 ± 2 nm (±2 nm is the variability among the samples), which is of the same order as the correlation length, ξ, estimated for the dense phases (34). The error bands in Figure 4D account for the slightly different entanglement spacing, chain dimensions, and concentrations in the different samples.

There are three characteristic times for chain relaxation (compare to eq. 6.106, 6.18, 6.19 in ref. (31)): $\tau_{\mathrm{e}} = \frac{a^6}{3\langle R^2\rangle^2 D}$ is the time at which the displacement of chain segments becomes comparable to the tube diameter, *a*; $\tau_{\mathrm{R_{tube}}} = \frac{a^2}{9\pi^2 D}$ is the time for chain relaxation within a tube; $\tau_{\mathrm{d}} = \frac{\langle R^2\rangle}{3\pi^2 D}$ is the disengagement time — the time required for a chain to disengage from the tube within which it was confined. In Figure S7B, we compared the experimental chain reconfiguration time and diffusion coefficient with the three characteristic times of an entangled chain with effective tube diameter of a = 4 nm and found that the chain relaxation that best describes the experimental results is τ_{d}. This observation suggests that the major contribution to end-to-end distance fluctuations is due to protein disengagement. Note that τ_{d} and τ_{R} defined for the Rouse model have the same dependence on *D*.

The viscosity of the polymer solution can also be obtained from the chain relaxation times by combining Eq. 21 with the three relations for τ_{e}, $\tau_{\mathrm{R_{tube}}}$, and τ_{d}. The finding that $\tau_r \approx \tau_d$ is valid both for the relation between chain reconfiguration time and diffusion coefficient (Eq. 4), and for the relation between chain reconfiguration time and viscosity (Eq. 5, Figure S7B). For consistency with section i), in Figure 4D and Figure S7B, we scaled τ_{e}, $\tau_{\mathrm{R_{tube}}}$, and τ_{d} relative to τ_{r} as described for τ_{R} in Eq. 15.

Molecular dynamics (MD) simulations

All-atom explicit solvent simulations of phase-separated ProTα-H1 in 8 mM KCl as well as ProTα-protamine in 8 mM KCl and 128 mM KCl were performed using the same simulation parameters as previously described for phase-separated ProTα-H1 at 128 mM KCl (12). In brief, we employed the Amber99SBws force field (35, 36) with the TIP4P/2005s water model (37, 38). The temperature was kept constant at 295.15 K using stochastic velocity rescaling (39) (τ = 1 ps), and the pressure was kept at 1 bar with a Parrinello-Rahman barostat (40). Long-range electrostatic interactions were modeled using the particle-mesh Ewald method (41) with a grid spacing of 0.12 nm and a real-space cut-off of 0.9 nm. Dispersion interactions and short-range repulsion were described by a Lennard-Jones potential with a cutoff at 0.9 nm. Bonds involving hydrogen atoms were constrained to their equilibrium lengths using the LINCS algorithm (42). Equations of motion were integrated with the leap-frog algorithm with a time step of 2 fs, with initial velocities taken from a Maxwell-Boltzmann distribution at 295.15 K. All simulations were performed using GROMACS (43) version 2021.5. We simulated the unlabeled variant of ProTα, since the droplets under experimental conditions had 1000-fold higher concentration of unlabeled than labeled ProTα.

To obtain the starting structure of phase-separated ProTα-H1 at the desired ion concentration of 8 mM KCl, and to ensure charge neutrality, we removed 2289 potassium and 2289 chloride ions from the snapshot at 5 μs of our previous phase-separated ProTα-H1 at 128 mM KCl (12). The simulation system contained 96 ProTα and 80 H1 molecules, 129 potassium and 241 chloride ions, and 899,220 water molecules, resulting in a simulation system of 3,996,354 particles. The free production run was 3.1 μs long, with a timestep of 2 fs, employing 36 nodes (each consisting of an Intel Xeon E5-2690 v3 processor with 12 cores and an NVIDIA Tesla P100 GPU at the Swiss National Supercomputing Centre) with a performance of ~35 ns/day, corresponding to ~3 months of supercomputer time. The first 2.1 μs were treated as system equilibration and not used for the analysis.

For the ProTα-protamine simulations, we used the same all-atom force field with explicit solvent described above, but to generate an initial structure for these simulations, a phase-separated system in slab configuration (44) was obtained with coarse-grained (CG) simulations, following the strategy described previously (12). For this initial setup only, we utilized the one-bead-per-residue model that was previously developed to study the 1:1 ProTα-H1 dimer (11). Briefly, the potential energy has the following form:

$$V = \frac{1}{2}\sum_{i<N} k_b\left(d_{ij} - d_{ij}^0\right)^2 + \frac{1}{2}\sum_{i<N-1} k_\theta\left(\theta_{ijk} - \theta_{ijk}^0\right)^2$$

$$+\sum_{i<N-2}\sum_{n=1}^{4}k_{i,n}(1+\cos(n\phi_{ijkl}-\delta_{i,n}))+\sum_{a<b}\frac{q_a q_b}{4\pi\epsilon_d\epsilon_0 d_{ab}}\exp\left[-\frac{d_{ab}}{\lambda_D}\right]$$
$$+\sum_{(a,b)}4\varepsilon_{pp}\left(\left(\frac{\sigma_{ab}}{d_{ab}}\right)^{12}-\left(\frac{\sigma_{ab}}{d_{ab}}\right)^{6}\right), \quad \text{Eq. 22}$$

where i, j, k, l denote consecutive residues. The first term represents the harmonic bond energy with force constant $k_b = 3.16 \times 10^5$ kJ. mol^{-1}nm^{-2}, and the second term represents the angle energy with force constant $k_\theta = 6.33 \times 10^2$ kJ mol^{-1}rad^{-2}; reference values for d_{ij}^0 and θ_{ijk}^0 were taken from an extended backbone structure (0.38 nm; $2\pi/3$ rad). The third term represents a sequence-based statistical torsion potential taken from the Go model of Karanicolas and Brooks (45), which was applied to all residues. The fourth term represents a screened coulomb potential, with Debye screening length λ_D, applied to all residues with non-zero charges q_i; ϵ_0 is the permittivity of free space; the dielectric constant, ϵ_d, was set to 80. The fifth term represents a generic short-range attractive potential applied to all residue pairs. This interaction is characterized by a contact distance $\sigma_{ab} = (\sigma_a + \sigma_b)/2$, where $\sigma_{a,b}$ are the residue diameters (all 0.6 nm) determined from residue volumes (46), and a contact energy ε_{pp}, which is the same for all residue pairs and was set to $0.16\ k_B T$, or ~ 0.40 kJ/mol^{-1}. The Debye length, λ_D, is given by

$$\lambda_D = \left(\frac{\epsilon_d\epsilon_0 k_B T}{2e^2 I}\right)^{1/2}, \quad \text{Eq. 23}$$

where k_B is the Boltzmann constant, T the temperature, e the elementary charge, and I the ionic strength.

Using this coarse-grained model, 96 ProTα and 197 protamine molecules (1:1 charge balance) in an initially extended configuration were placed on a rectangular grid in a 60-nm cubic box, and the energy of the system was minimized with the steepest-descent algorithm. The system was further relaxed in a short NVT run at 500 K and an implicit ionic strength of 500 mM. In the next step, the box edge was decreased to 22.41 nm in a 56-ps NPT run with reference pressure set to 20 bar to obtain an average protein density close to that of the dense phase in experiment. The system configuration was further randomized via a 1.5-μs NVT run (using a 10-fs time step) at 500 K and an implicit ionic strength of 500 mM to ensure relatively uniform protein density in the box. Each chain from the final CG structure was independently reconstructed in all-atom form using a lookup table from fragments drawn from the PDB, as implemented in Pulchra (46). Side-chain clashes in the all-atom representation were eliminated via a short Monte Carlo simulation with CAMPARI (47) using the ABSINTH energy function (47), in which only the side chains were allowed to move. Due to the large number of proteins and the relatively high density of proteins in the box, the first CAMPARI moves were performed using a soft-core Lennard-Jones (LJ) potential, which has an energy cap, thus avoiding the very large energies resulting from the exclusion in the first Monte Carlo moves at the beginning of the CAMPARI simulation. Subsequently, the soft-core LJ potential was gradually transformed into a global soft-core form by increasing the CAMPARI parameters FMCSC_FEG_IPP and FMCSC_FEG_ATTLJ. The following pairs of FMCSC_FEG_IPP and FMCSC_FEG_ATTLJ values were used: 0.5 and 0.35, 0.7 and 0.6, 0.9 and 0.85, 0.95 and 0.92, 0.98 and 0.96, 0.99 and 0.97, 0.9925 and 0.975, 0.995 and 0.99. Taking the relaxed configuration obtained with CAMPARI, the box edge was extended to 41 nm in the Z direction, and the resulting system was set up with the all-atom Amber ff99sbws protein force field (35, 36) in GROMACS and energy-minimized using the steepest-descent algorithm. To eliminate any non-proline cis bonds that might have emerged during all-atom reconstruction, we ran a short simulation in vacuum with the dielectric constant set to 80 with periodic boundaries, using a version of the force field that strongly favors trans peptide bonds (44) and applying weak position restraints to the protein backbone atoms and dihedral angles (5 kJ/mol/rad).

Subsequently, the simulation box was filled with TIP4P/2005s water[44] and energy-minimized. Up to this point, the same setup was used for both 8 mM and 128 mM ProTα-protamine simulations. In the next step, 1587 potassium and 1596 chloride ions were added to the simulation box for ProTα-protamine at 128 mM KCl (2,612,851 particles in total), and 99 potassium and 108 chloride ions were added to the simulation box for ProTα-protamine at 8 mM KCl (2,621,779 particles in total), to match the ionic strength of the buffer used in the experiments and to ensure charge neutrality. In the next step, both systems were again energy-minimized, and a 10-ns MD run was performed with strong position restraints on protein backbone atoms (10^5 kJ mol^{-1} nm^{-2}) to stabilize the trans isomer for any peptide bonds that had isomerized in the previous step. The final structures of these 10-ns runs with backbone restraints were used for the production runs (without restraints), using GROMACS (43) versions 2021.5 and 2024.1. The free production runs at 128 mM and 8 mM KCl were 4 µs long, with a timestep of 2 fs. For the first 2.5 µs of both simulations, we employed 48 nodes (each node consisting of an Intel Xeon E5-2690 v3 processor with 12 cores and an NVIDIA Tesla P100 GPU at the Swiss National Supercomputing Centre) with a performance of ~63 ns/day, corresponding to ~40 days of supercomputer time. The last 1.5 µs of both simulations were run on NVIDIA Grace-Hopper (GH200) nodes at the Swiss National Supercomputing Centre, using 192 CPUs and 4 GPUs per simulation, with a performance of ~62 ns/day, corresponding to ~24 days of supercomputer time. The first 3 µs of both simulations were treated as system equilibration (given the change in protein density, see Fig. S4B) and not used for analysis.

Analysis of MD simulations

For chain dynamics with relaxation times much shorter than the total simulation time, distance correlation functions can be calculated directly from the simulations and compared with experiment, as demonstrated previously for complex coacervates of H1 + ProTα (12). For the simulations of protamine + ProTα, however, chain relaxation is too slow for this approach to be reliable. To estimate long-timescale dynamics and correlation functions from the condensate trajectories also for these cases, the dynamics of each ProTα chain were described as one-dimensional diffusion along the coordinate defined by the separation in space, *r*, of the residues that were dye-labeled in experiment (here we use the distance between Cα atoms of residues 58 and 112) (Figure S9). That is, the separation is considered to diffuse on a free energy surface, $F(r)$, with a diffusion coefficient D_F (more generally, position-dependent $D_F(r)$), whose parameters we determine from the simulations. This distance coordinate is first discretized into *b* bins of equal width, from which the number of transitions, $N_{ji}(\Delta t)$, from bin *i* to bin *j* after a lag Δt during the simulations is counted. These statistics are combined from all ProTα chains, considering that they are expected to be indistinguishable. Discretized free energies and diffusion coefficients were optimized via Monte Carlo simulations using the likelihood function

$$\ln L = \sum_{i,j} N_{ji}(\Delta t) \ln p(j, t + \Delta t | i, t), \qquad \text{Eq. 24}$$

where the propagators $p(j, t + \Delta t | i, t)$ describing the conditional probability of being in bin *j* at time Δt after having been in bin *i* are obtained from the discretized diffusion model as previously described (48, 49): in short, the discretized dynamics is mapped to a chemical kinetics scheme describing evolution of populations in the bins, $\dot{\mathbf{P}}(t) = \mathbf{KP}(t)$, where $\mathbf{P}(t)$ is the vector of the bin populations at time *t*, and **K** is a rate matrix derived from the diffusion coefficient(s) D_F (or $D_{F,i}$ for position-dependent D_F) and free energies F_i associated with each bin according to the scheme of Bicout and Szabo (48, 50). The propagators are then given by $p(j, t + \Delta t | i, t) = (\exp[\Delta t\mathbf{K}])_{ji}$. In estimating the most probable parameters from the data, a uniform prior is used for the diffusivities and free energies. The statistical error on the derived parameters is determined by generating synthetic data sets with the same number *M* of individual distance trajectories $r(t)$ as the original, specifically by choosing *M* trajectories randomly with replacement, and refitting the model. The error is taken as the standard deviation of the parameters across all synthetic data sets (51).

We can compute the normalized correlation functions directly from the discretized diffusion model via (52)

$$C(t) = \frac{\sum_{n=2}^{b}(\mathbf{r}\cdot\boldsymbol{\psi}_n^R)^2 \exp[\lambda_n t]}{\sum_{n=2}^{b}(\mathbf{r}\cdot\boldsymbol{\psi}_n^R)^2}, \qquad \text{Eq. 25}$$

where the elements of **r** are the centers of each bin on the distance coordinate, $\boldsymbol{\psi}_n^R$ is the *n*th right eigenvector of **K** ($\boldsymbol{\psi}_1^R$ is the stationary eigenvector), and λ_n is the *n*th eigenvalue. Similarly, the correlation times are given by

$$\tau_c = -\frac{\sum_{n=2}^{b}(\mathbf{r}\cdot\boldsymbol{\psi}_n^R)^2 \lambda_n^{-1}}{\sum_{n=2}^{b}(\mathbf{r}\cdot\boldsymbol{\psi}_n^R)^2}. \qquad \text{Eq. 26}$$

Errors in correlation functions and correlation times were estimated using the same procedure as for the diffusion model parameters. In our application of the method to the condensate trajectories, we have used 30 equal-width bins between 2 and 10 nm, and a lag time of 200 ns. For consistency, in the present work we use this analysis for all condensates, and for H1-ProTα, where the correlation function can also be obtained directly from the intrachain distance (12), the resulting reconfiguration times are similar.

A key assumption of this method is that the dynamics is, indeed, well approximated as diffusive after the chosen lag time Δt. If this is true, then the model should become independent of lag time beyond this point. To assess this effect, we computed the correlation time as a function of the lag time and observed that after lag times of around 200 ns it appears to be converging toward a limiting value (Figure S9D). One challenge for using even longer lag times is the limited length of the simulations, resulting in insufficient statistically independent observations. Separately from the statistical error estimate described above, we also estimated the systematic error associated with the choice of lag time by using correlation times computed at 100 and 300 ns lag times as lower and upper error bars, respectively. A second assumption we have made is that the diffusion coefficient should be uniform, i.e. not dependent on the position on the distance coordinate. This was motivated by our finding that using an explicitly position-dependent diffusion coefficient resulted in very little position dependence, as demonstrated in Figure S9B. Although this conclusion differs from some earlier work (53), this is most likely because we do not significantly sample the very short distances where the position dependence of D_F emerged in that study.

The average number of H1 or protamine molecules that simultaneously interact with a single ProTα chain, as well as the average number of ProTα chains that simultaneously interact with a single H1/protamine molecule (Fig. 3d) in the dense-phase simulation were quantified by calculating the minimum distance between each ProTα and each H1/protamine for each simulation snapshot. The two molecules were considered to be in contact if the minimum distance between any two of their Cα atoms was within 1 nm. Distances between Cα atoms were used instead of the commonly used distances between all atoms of the residues to facilitate the large calculations. The 1-nm cutoff between the Cα atoms of two residues yields similar results as the commonly used 0.6-nm cutoff for interactions between any pair atoms from the two residues (43). The same contact definition was employed when calculating residue-residue contacts (Fig. 3e): Two residues were considered to be in contact if the distance between their Cα atoms was within 1 nm.

Lifetimes of residue-residue contacts were calculated by a transition-based or core-state approach (12, 54). For each pair of residues, a contact was based on the shortest distance between any pair of heavy atoms, one from each residue. Starting from an unformed contact, contact formation was defined to occur when this distance dropped below 0.38 nm; an existing contact was considered to remain formed until the distance increased to more than 0.8 nm (54). Average lifetimes of each residue-residue contact were calculated by dividing the total bound time by the total number of contact breaking events for that contact. Intra-chain contacts were not included in the analysis. Average lifetimes of each pair of ProTα-H1 and ProTα-protamine residues (averaged over the different combinations of ProTα and H1/protamine chains that the two residues could be part of) were calculated by dividing the total contact time (summed over all combinations of ProTα

and H1/protamine chains) of a specific residue pair by the total number of the contact breaking events for the same residues (summed over the same combinations of chains). Similarly, to calculate average lifetimes of residue-residue contacts according to the residue type, we first identified all contacts involving a particular pair of residue types, in which one residue was from the ProTα chain and the second was from either H1/protamine or ProTα. Subsequently, the average lifetime of that residue-residue combination was calculated by dividing the total bound time by the total number of contact breaking events for the contacts involving those residue types. Excess populations of contacts between specific types of residues were determined by dividing the average number of observed contacts for a pair of residue types by the value that would be expected if residues paired randomly in a mean-field approximation. The average number of contacts for a pair of residue types was calculated as a sum of all times that residues of those types were in contact, divided by the simulation length. The expected average number of contacts between two residue types (type 1 and 2) was calculated as $N f(1) f(2)$, where N is the average total number of contacts, and $f(1)$ and $f(2)$ are the fraction of residues of type 1 and 2, respectively.

The mean squared displacement (MSD) of individual residues within ProTα molecules were calculated as a function of delay time using the Gromacs function *gmx msd*. MSD curves of each ProTα residue for each of the 96 chains in simulations with protamines as well as in simulation with H1 at 8 mM KCl were calculated from the last microsecond of each of the simulations, using residue coordinates every 100 ps. MSD curves of each ProTα residue for each of the 96 chains in ProTα-H1 simulation at 128 mM KCl were calculated previously in four 1-μs blocks, using residue coordinates every 100 ps.

All of these analyses are consistent with the previous reports in Galvanetto *et al*. (12).

Distance distributions of the closest and second-closest residues (Figure 3H) were computed between all residues of all polypeptides in the simulations. The distances were considered between specific atoms in the side chains: Cδ for glutamate; Cγ for aspartate; Nζ for lysine; Cζ for arginine; and Cβ for alanine. The distributions shown in Figure 3H are averages of 100 structures taken every 1 ns. The percentages of contacts that are exchanged, broken and reformed, or remain unbroken (Figure 3K) were determined by first identifying the lysines in H1 and the glutamates in ProTα in the H1–ProTα slab at 128 mM KCl that form a close contact at time t_0, defined as being within 0.43 nm, corresponding to the sharp peak in the distribution of closest lysine contacts (Figure 3H, top panel). These contacts were then tracked for 100 ns and the fraction of contacts that separated beyond 0.43 nm during this time was quantified, at which point another lysine forms a closer contact. Additionally, we quantified the fraction of contacts that remain intact for the entire 100 ns and the fraction that, while not continuously maintained, breaks and reforms between the same two residues. The same analysis was performed for arginine (instead of lysine) in the protamine–ProTα dense phase at 128 mM KCl, using a distance threshold of 0.53 nm, corresponding to the peak in the distribution of closest arginine contacts (Figure 3H, middle panel).

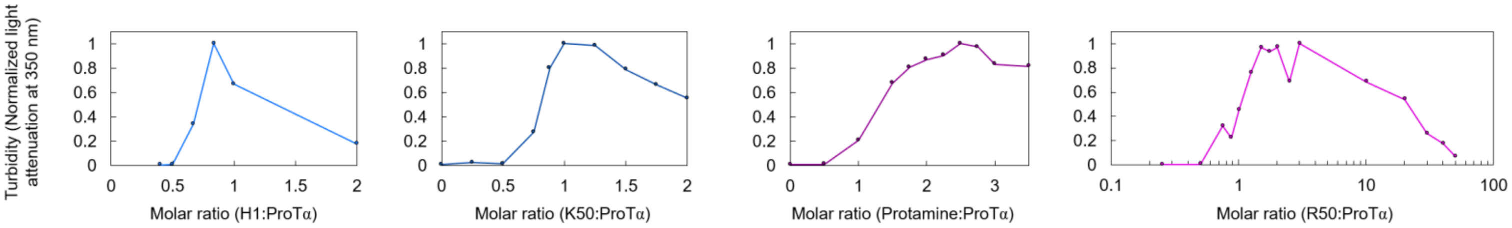

Figure S1. Phase separation is most pronounced for protein mixtures near charge balance. The extent of droplet formation was assessed using turbidity at a constant concentration of 10 µM ProTα and varying amounts of its polycationic partners at 120 mM KCl. Maximal phase separation was observed close to molar ratios, λ, where the charges of the two polymers balance (net charges: ProTα, -44; H1, +53; K50, +50; protamine, +22; R50, +50). From left to right, $\lambda_{H1:ProT\alpha}$ = 44:53 ≈ 0.8:1, $\lambda_{K50:ProT\alpha}$ = 44:50 ≈ 0.9:1, $\lambda_{protamine:ProT\alpha}$ = 44:22 = 2:1, $\lambda_{R50:ProT\alpha}$ = 44:50 ≈ 0.9:1. We note that the arginine-rich samples (protamine and R50) tend to phase-separate even with an excess of the polycationic partner, reflecting the complex interactions of arginine beyond Coulomb interactions (55, 56).

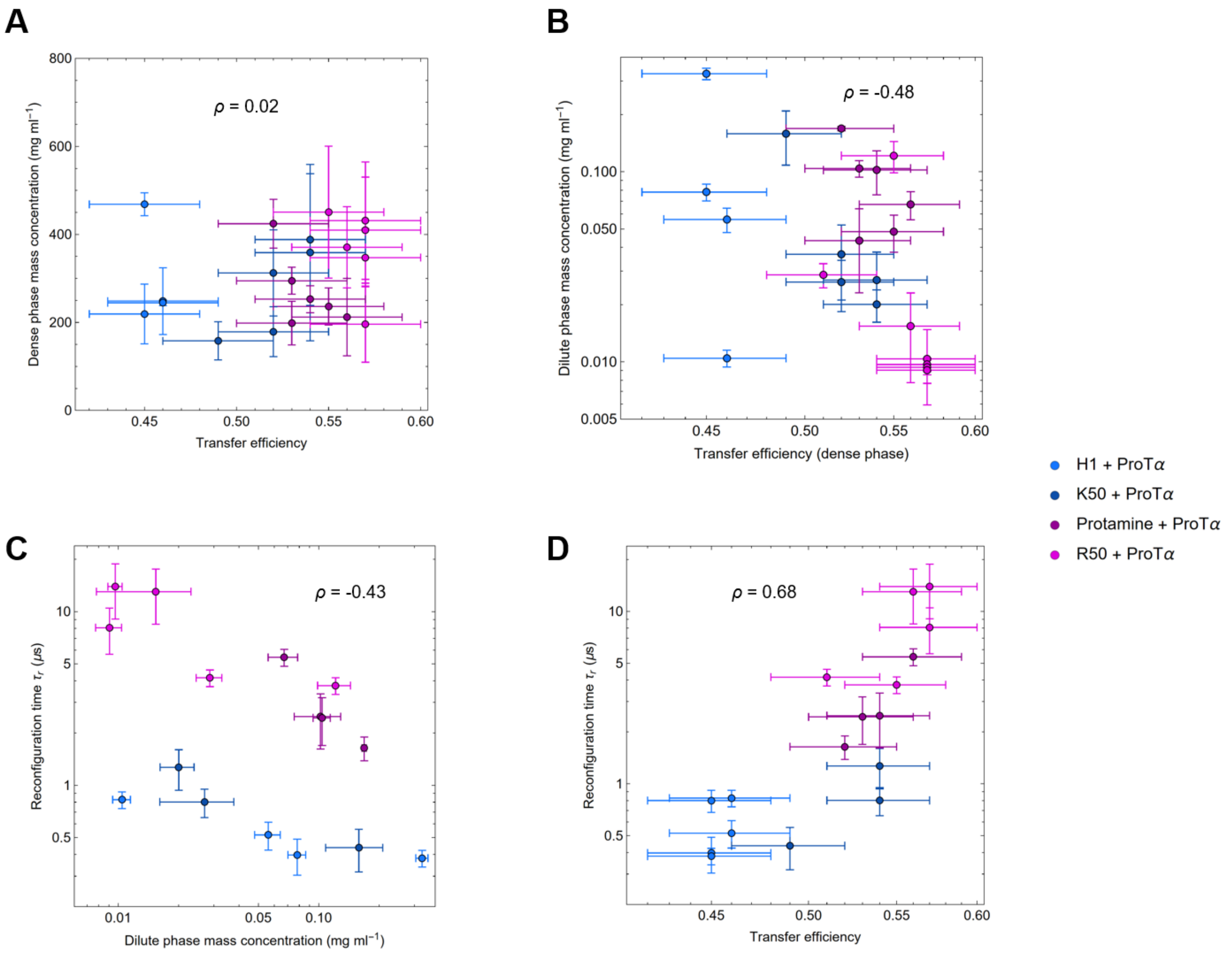

Figure S2. Correlations between thermodynamic and dynamic quantities in the dense and dilute phases. (**A**) ProTα transfer efficiency *vs* dense phase protein concentration shows no correlation. (**B**) Dense-phase ProTα transfer efficiency *vs* dilute-phase protein concentration shows a slight anticorrelation, indicating that both thermodynamic quantities are proxies for the interaction strength between polymers: the stronger the interaction, the lower the dilute phase concentration and the smaller the chain dimensions (13). (**C**) Correlations between chain reconfiguration time, τ_r, and dilute phase mass concentration, and (**D**) between τ_r and transfer efficiency indicate relations between the molecular dynamics within condensates and the intermolecular interactions of the systems, as proposed by An *et al*. (57). ρ are the Pearson correlation coefficients.

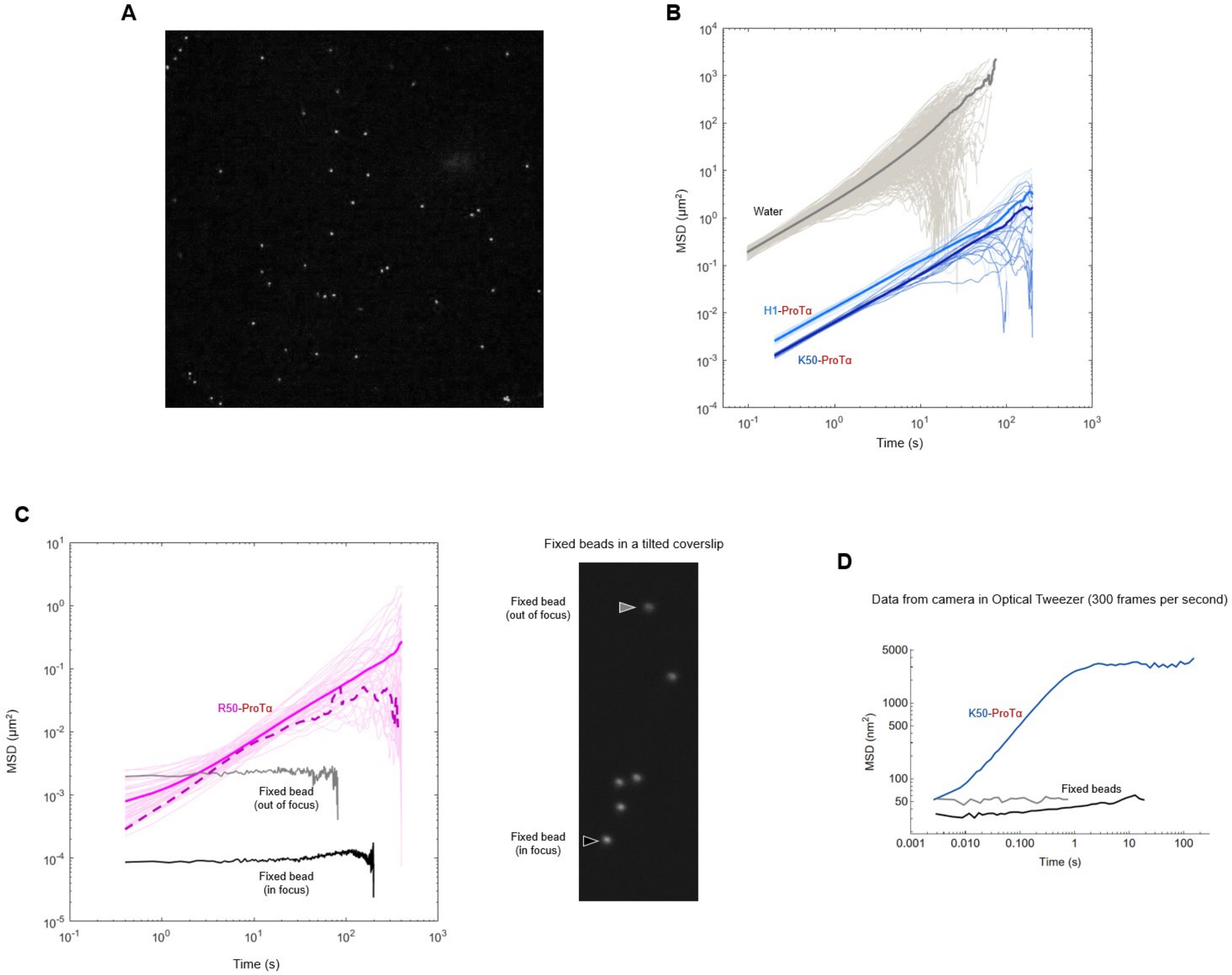

Figure S3. Passive microrheology shows Brownian diffusion of beads in the dense phase down to the millisecond timescale. (**A**) Example of a fluorescence micrograph of 500-nm beads in a K50-ProTα droplet (image size: 150x150 μm). (**B**) Mean-squared displacement (MSD) of individual 500-nm beads and their averages (solid thick lines) in water, in K50-ProTα dense phase in 60 mM KCl, and in H1-ProTα dense phase in 120 mM KCl, show Brownian diffusion. (**C**) MSD of 500-nm beads in R50-ProTα dense phase in 90 mM KCl (right) apparently deviates from Brownian diffusion at short times, which might be mistaken to suggest the approach of the elastic plateau (18). Further examination of the MSD of beads fixed on a cover slide at different positions relative to the focal plane (left) indicates that this deviation is an artifact due to the limited precision in determining the position of the beads. A stricter threshold for automatic bead identification can reduce this artifact (dashed line), but it also reduces the number and length of individual trajectories, making viscosity determination problematic over longer time periods. (**D**) The MSD as a function of time for a single polystyrene bead (1 μm diameter) trapped in the dense phase of K50-ProTα with optical tweezers also appears to change slope at short times. Similarly, the deviation observed for MSD values <100 nm^2 is likely to be caused by the limited precision in determining the position of the beads. (Tracking was performed from brightfield images taken with maximum LED illumination, see Methods.)

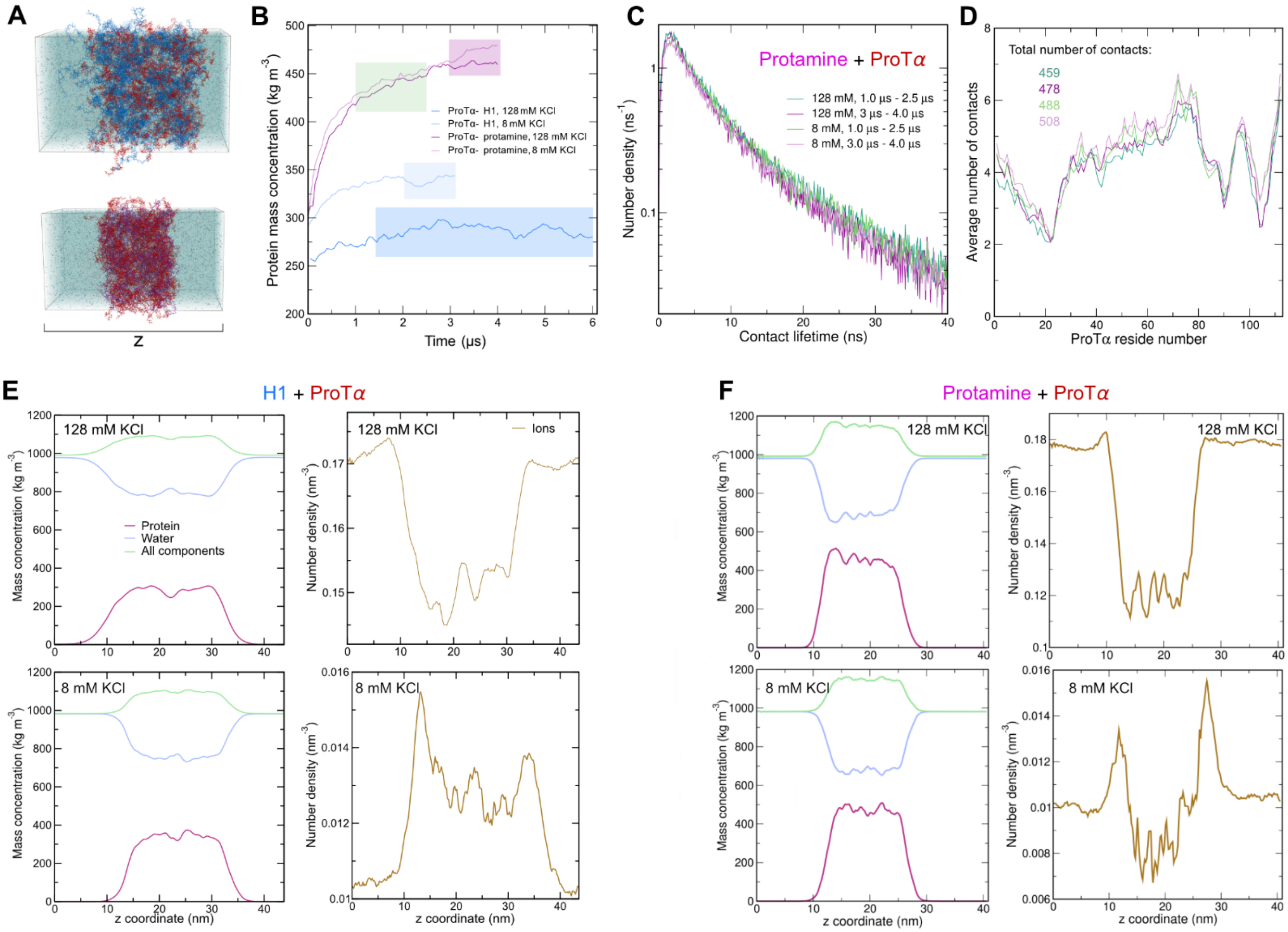

Figure S4. Mass concentrations and ion distributions in MD simulations. (**A**) Illustrations of H1–ProTα and protamine–ProTα simulations in slab geometries. Densities were calculated along the z-axis. (**B**) Protein concentration in the dense phases as a function of time, calculated in 50-ns blocks. The parts of the simulations where the protein density strongly increases with time were treated as equilibration and omitted from the analysis. The parts of the simulations that were analyzed are highlighted by the shaded boxes. (**C**-**D**) The effect of protein density differences on the contact lifetimes (E) and on the number of contacts per residue (F) is small. Two segments of the protamine–ProTα trajectories were analyzed, spanning 1 to 2.5 µs and 3 to 4 µs, respectively (indicated by the green and purple boxes in panel B). Despite the difference in protein density of ~6% between the two time windows, their lifetime distributions are very similar, while the average contact lifetimes increase slightly (by ~10%), mostly due to a relatively small number of very long contacts. The total number of contacts between ProTα and protamine (D) increases by ~4%, consistent with the slight increase in protein density. **(E–F)** Mass concentrations of protein, water, all components (protein, water, and ions; left panels), and number density of ions (right panels) along the z-axis of the simulation box in the four different simulations. The water density in the protamine–ProTα simulations is lower than the water density in the H1–ProTα simulations, which is consistent with the higher protein density observed in the protamine–ProTα simulations. For the simulations with a total salt concentration of 128 mM KCl, ion concentrations within the dense phase are decreased relative to the dilute phase in both the H1–ProTα and protamine–ProTα

simulations, in parallel with the decrease in water content within the dense phases. Conversely, for the simulations with a total salt concentration of 8 mM KCl, ion concentrations are increased in the dense phase relative to the dilute phase in both the H1–ProTα and protamine–ProTα simulations. It is worth noting that a dependence of salt partitioning on the salt concentration has also been observed for synthetic complex coacervates (58).

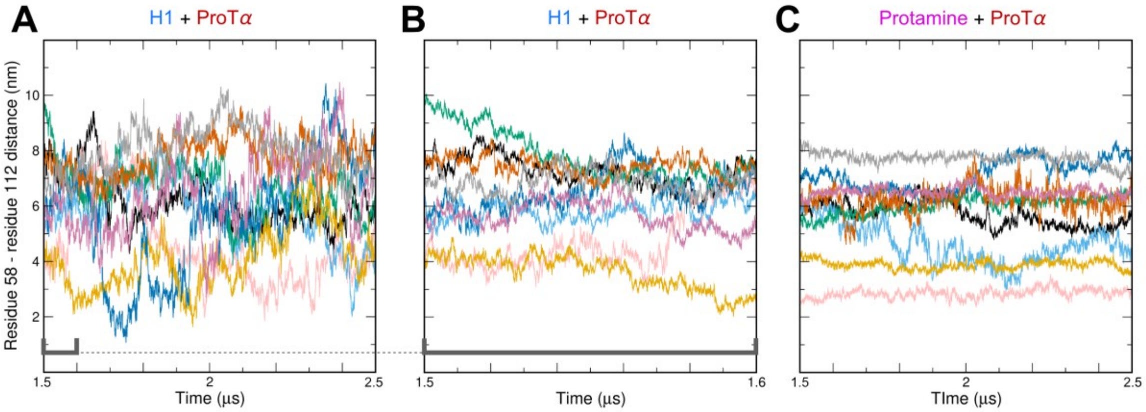

Figure S5. Comparison of ProTα chain dynamics in H1–ProTα and protamine–ProTα dense phase at 128 mM KCl from MD simulations. (**A**) Examples of intrachain distance fluctuations between residues 58 and 112 for 9 of the 96 ProTα chains (chain 10, 20, ... , 90) in the H1–ProTα dense phase during 1 µs. (**B**) Same as **A**, but illustrated for the first 0.1 µs. (**C**) Examples of intrachain distance fluctuations between residues 58 and 112 for 9 of the 96 ProTα chains in the protamine–ProTα dense phase over 1 µs are qualitatively comparable to the distance fluctuations in the H1–ProTα slab during 0.1 µs, illustrating the ~10-fold slower chain dynamics in the protamine–ProTα dense phase, which is also reflected in the chain reconfiguration times (Figures 3 and S9).

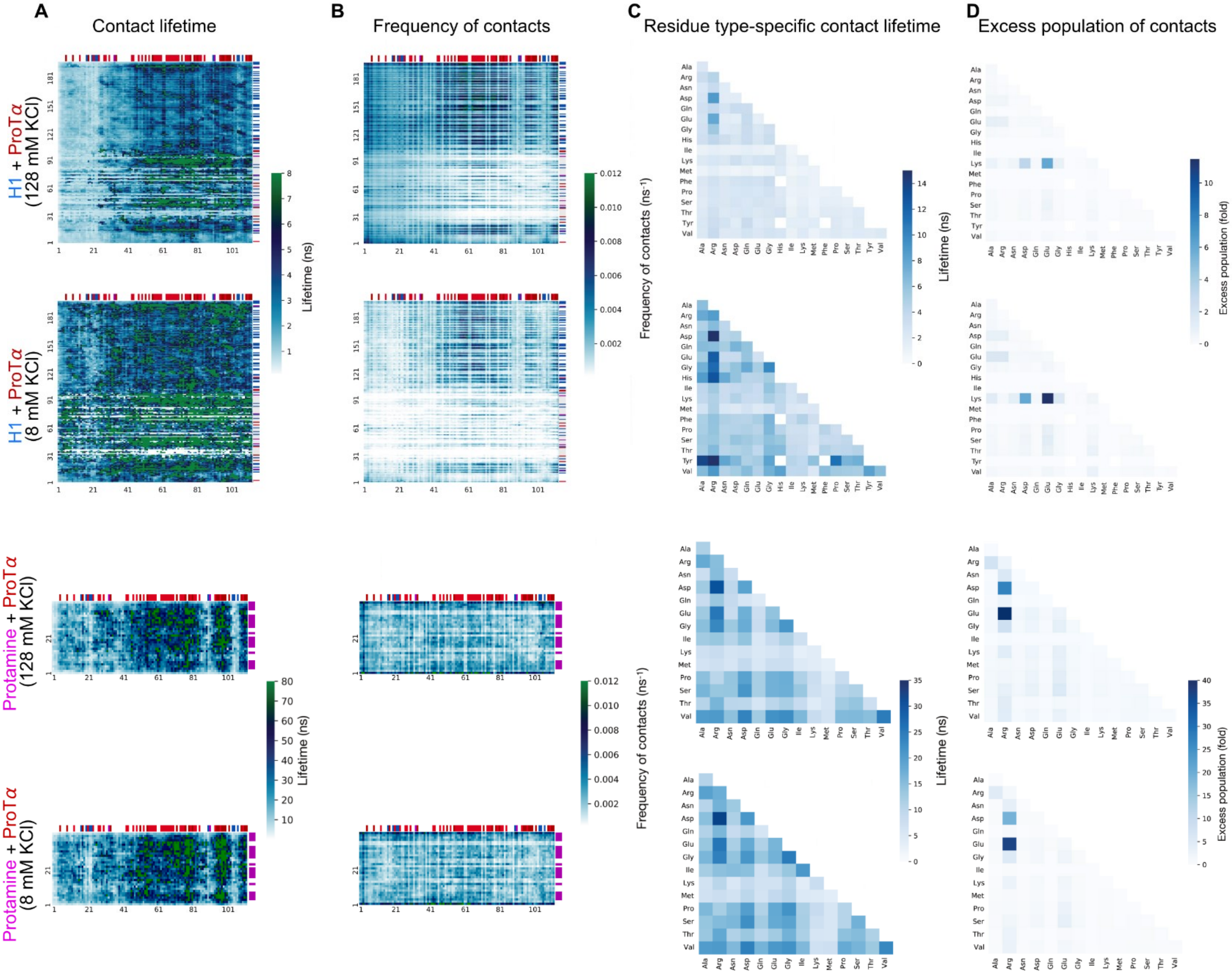

Charged amino acids

- Glutamate (E) −
- Aspartate (D) −
- Lysine (K) +
- Arginine (R) +

Number of residues

	Ala	Arg	Asn	Asp	Cys	Gln	Glu	Gly	His	Ile	Leu	Lys	Met	Phe	Pro	Ser	Thr	Trp	Tyr	Val
Protamine	0	21	0	0	0	0	0	2	0	0	0	0	1	0	3	4	0	0	0	2
H1	29	6	3	4	0	5	5	7	2	7	5	56	1	2	15	20	12	0	3	11
ProTα	11	2	6	19	0	2	34	10	0	1	1	8	1	0	2	4	6	0	0	5

Figure S6. Contact lifetime heatmaps from MD simulations. (**A**) Average lifetime of residue-residue contacts, calculated by considering all instances of these residues across all chains. Numbers on the bottom and left of the heatmaps denote the residue numbers of ProTα and H1 or protamine, respectively. Note that H1 contains a folded globular domain (GD) between residues 22 and 96. Bars at the top and right of the plots denote charged residues (color code as in Figure 1A). (**B**) Frequency of contacts (defined as the number of new contacts made by one ProTα residue per nanosecond). Panels **A** and **B** indicate that: (i) decreasing salt concentration increases the lifetime of residue-residue contacts (see also Figure 3F); (ii) contact times in arginine-rich droplets (Protamine–ProTα) are longer than in lysine-rich droplets (H1–ProTα) (see also Figure 3F); (iii)

charge-charge contacts are the most frequent but the most short-lived contacts in the lysine-rich condensates, whereas charge-charge contacts are both the most frequent and the longest-lived in the arginine-rich condensates, reflecting the propensity of arginine to form multivalent contacts (see also Figure 3J). (**C**) Average lifetimes of residue-residue contacts classified by residue type. Residue pairs that are never observed (white squares) and extremely long-lived pairs (dark blue) typically correspond to residue types that are rare in the ProTα and H1/protamine sequences. (**D**) Excess populations of contacts between specific types of residues (determined by dividing the average number of observed contacts for a pair of residue types by the value that would be expected if residues paired randomly in a mean-field approximation, see Methods). The large excess of contacts between charged residues indicates that their interactions are the most favorable in both lysine- and arginine-rich condensates. Although these contacts are the most frequent, their lifetimes are in the same range as those of other residue pairs (see **C**). In addition, the excess of charged residue interactions is more pronounced in arginine-rich condensates than in lysine-rich condensates, in line with the propensity of arginine to form multivalent contacts (59–62).

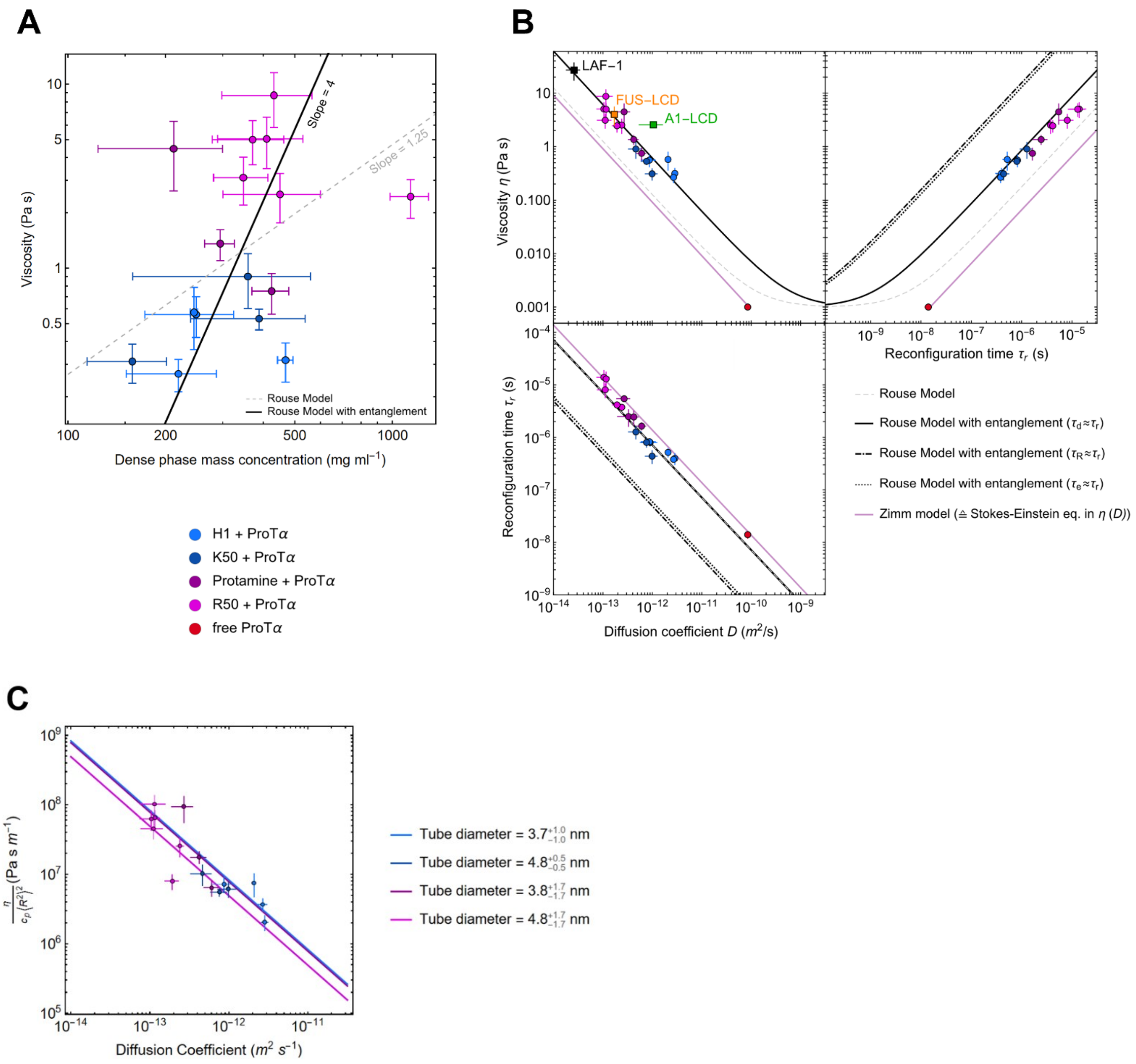

Figure S7. Comparison of experimental data with different polymer models. (**A**) The dependence of viscosity, η, on the dense-phase concentration, c_p, is better described by the Rouse theory with entanglement, which predicts a viscosity dependence between $\eta \propto {c_p}^{3.75}$ (see equation 7.65 in ref. (31)) and $\eta \propto {c_p}^{4.25}$ (63) (total least squares of $Log(\eta/1\ Pa\ s)$ and $Log(\frac{c_p}{1} mg\ ml^{-1})$ = 2.7 for a slope of 4), rather than by the Rouse theory without entanglement, which predicts $\eta \propto {c_p}^{1.25}$ (63) (total least squares = 6.7 for a slope of 1.25). (**B**) Comparison of the experimental viscosity, diffusion coefficient, and chain reconfiguration time with the predictions of the Rouse model, the Zimm model, and the Rouse model with entanglement from Equations 1-5 and 13-21

(see Methods). The viscosities and diffusion coefficients of LAF-1 (64), A1-LCD (13, 65), and FUS-LCD (66, 67) are from previous reports. The average tube diameter used in the Rouse model with entanglement is obtained as shown in **C** (a = 4±2 nm). The Rouse model with entanglement predicts three different chain relaxation times: $\tau_{\mathrm{e}} = \frac{a^6}{3\langle R^2\rangle^2 D}$ is the time at which the displacement of chain segments becomes comparable to the effective tube diameter, a; $\tau_{\mathrm{R_{tube}}} = \frac{a^2}{9\pi^2 D}$ is the time for chain relaxation within a tube; $\tau_{\mathrm{d}} = \frac{\langle R^2\rangle}{3\pi^2 D}$ is the disengagement time — the time required for a chain to disengage from the tube within it was confined. Since it is not obvious which of these relaxation times corresponds to the experimental reconfiguration time τ_{r} (i.e. the measurement of the end-to-end fluctuations of single chains with FRET-based nsFCS), we compare the three models (black lines). The chain relaxation that best describes the experimental results is τ_{d}, suggesting that the experimentally observed end-to-end distance fluctuations are dominated by chain disengagement. (**C**) The reduced viscosity, $\frac{\eta}{c_p\langle R^2\rangle^2}$, allows us to evaluate the relation between viscosity and diffusion coefficient *D* to obtain the best estimate for the tube diameter, a (see Eq. 4), taking into account the contribution from the slightly different chain dimensions, $\langle R^2\rangle$, and protein concentrations, c_p, in the individual samples. The solid lines correspond to the relation $\frac{\eta}{c_p\langle R^2\rangle^2} = \frac{k_B T}{36\,D}\frac{1}{a^2} + \frac{\eta_s}{c_p\langle R^2\rangle^2}$, where the tube diameters, a, were calculated for each individual sample as $a = \sqrt{\frac{k_B T}{36\,D}\frac{c_p\langle R^2\rangle^2}{\eta}}$. The mean values and standard deviations of a for the individual samples are reported in the legend. For the comparison with other models, we used the average value a = 4±2 nm, which is in agreement with the value independently estimated using only c_p and $\langle R^2\rangle$ following ref. (23).

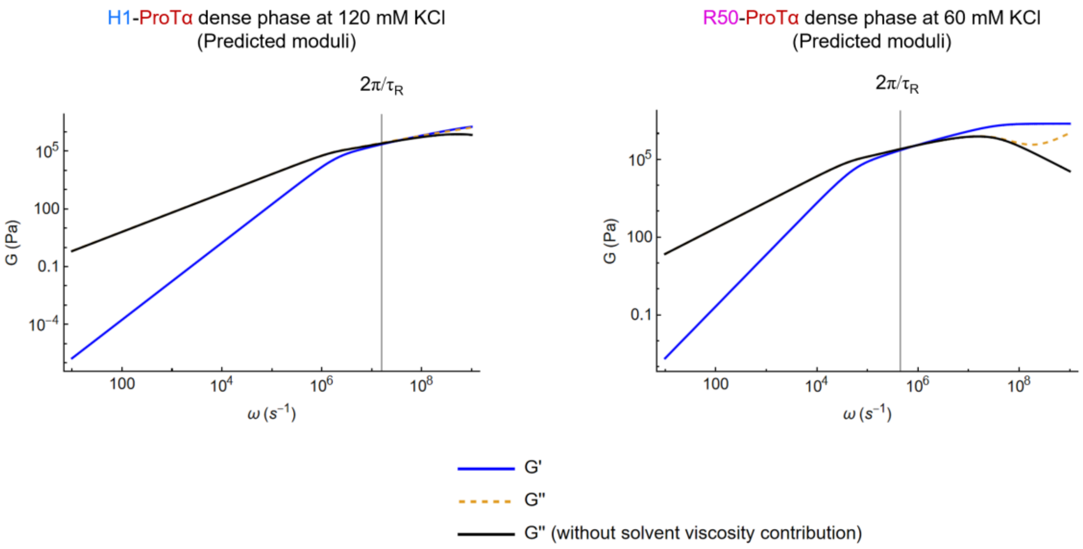

Figure S8. Theoretical storage and loss moduli of the samples with the fastest (left) and slowest (right) chain reconfiguration time, τ_r. The Rouse model (27) can be used to calculate the storage modulus $G'(\omega) = c_p k_B T \sum_p \frac{\omega^2 \tau_p{}^2}{1+\omega^2 \tau_p{}^2}$ and the loss modulus $G''(\omega) = \omega\eta_s + c_p k_B T \sum_p \frac{\omega \tau_p}{1+\omega^2 \tau_p{}^2}$ of a polymer solution with a concentration of chains, c_p, and solvent viscosity, η_s, where ω is the angular frequency, and τ_p is the relaxation time of the *p*-th Rouse mode ($\tau_p = \frac{\tau_\mathrm{R}}{p^2}$, where the longest relaxation mode, $\tau_1 \triangleq \tau_\mathrm{R}$, is related to the reconfiguration time of the experimentally observed chain segment by the Makarov relation (32), $\tau_\mathrm{R} = \frac{\tau_r}{0.27}$, see Methods). This theory has previously been shown to accurately describe experimental data on synthetic polymers (68). We note that even in the sample with the slowest reconfiguration time investigated here (R50-ProTα at 60 mM KCl), the predicted crossover frequency, $\frac{2\pi}{\tau_\mathrm{R}}$, is in the megahertz range, which is difficult to access using conventional rheological methods (69). Similarly, in the case of Rouse theory with entanglement, the crossover frequency is expected to be the inverse of the disengagement time, τ_d, (18, 31) which corresponds to the experimental chain reconfiguration time, τ_r (see Figure S7B).

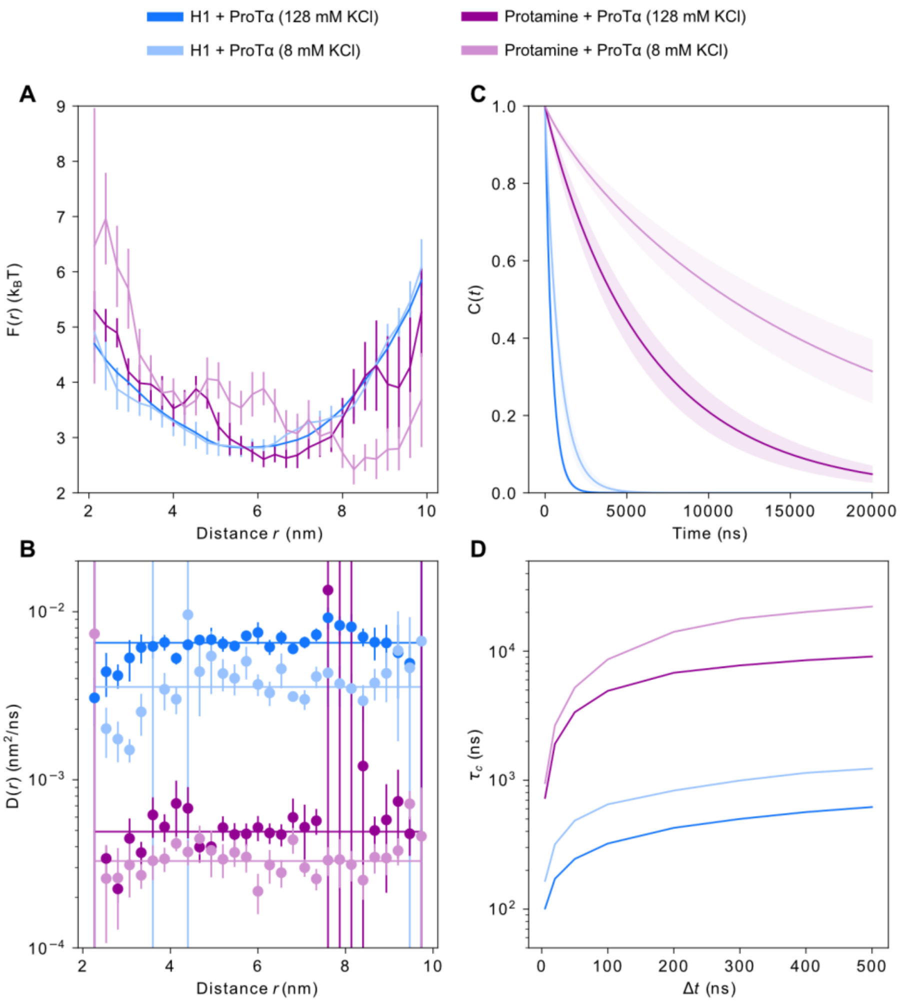

Figure S9. Computing distance decorrelation times from simulation using a diffusion model. For each system, the dynamics of the 58-112 distance is modeled using a discretized diffusion model described by (**A**) free energies, *F*(*r*), and (**B**) diffusion coefficients, *D*(*r*), determined from the molecular simulations. Allowing position-dependent diffusion coefficients (symbols in (**B**)) shows that constant *D* (horizontal lines in (**B**)) is a good approximation. The model allows properties beyond the ~1 μs time scale of the equilibrated portion of most of the simulations to be estimated, such as (**C**) correlation functions and (**D**) correlation times. Based on the convergence of correlation time, τ_c, with lag time, Δt, in (**D**), a lag time of 200 ns was chosen for all systems and was used in the models for (**A**)-(**C**). Note that for the more rapidly relaxing H1+ProTα condensates, the distance correlations can be calculated directly from the simulation trajectories without using a diffusion model and yield similar values.

ProTα (unlabeled)	GPMSDAAVDTSSEITTKDLKEKKEVVEEAENGRDAPANGNANEENG<br>EQEADNEVDEEEEEEGGEEEEEEEEEGDGEEEDGDEDEEAESATGKR<br>AAEDDEDDDVDTKKQKTDEDD
ProTαC (56C/110C labeled)	GPSDAAVDTSSEITTKDLKEKKEVVEEAENGRDAPANGNAENEENG<br>EQEADNEVDEE**C**EEGGEEEEEEEEEGDGEEEDGDEDEEAESATGKR<br>AAEDDEDDDVDTKKQKTDED**C**
H1 (unlabeled)	TENSTSAPAAKPKRAKASKKSTDHPKYSDMIVAAIQAEKNRAGSSRQ<br>SIQKYIKSHYKVGENADSQIKLSIKRLVTTGVLKQTKGVGASGSFRLAK<br>SDEPKKSVAFKKTKKEIKKVATPKKASKPKKAASKAPTKKPKATPVKK<br>AKKKLAATPKKAKKPKTVKAKPVKASKPKKAKPVKPKAKSSAKRAGK<br>KK
Protamine (unlabeled)*	MPRRRRSSSRPVRRRRRPRVSRRRRRRGGRRRR
Poly-L-lysine 50	KKKKKKKKKKKKKKKKKKKKKKKKKKKKKKKKKKKKKKKKKKKKKKK<br>KKK
Poly-L-arginine 50	RRRRRRRRRRRRRRRRRRRRRRRRRRRRRRRRRRRRRRRRRRR<br>RRRRRRR

Table S1. Amino acid sequences of polypeptides used in experiments and simulations. Cys residues introduced for labeling are indicated in bold. Unlabeled ProTα is a variant of human ProTα isoform 2, while ProTα 56C/110C is a variant of isoform 1 (11). The isoforms differ by a single Glu at position 39. *We note that the sequence given here and used in the simulations is a main component of the commercially available protamine we used (Sigma-Aldrich, product number P4020), but other components with similar lengths and amino acid composition are present in the preparation.

Video S1 (separate file)

(Left) All-atom explicit-solvent simulation of the ProTα–H1 condensate (total time 1 μs). One ProTα chain is highlighted in red (chain 60), and four interacting H1 chains are shown in different shades of blue. Other surrounding ProTα and H1 chains are shown semi-transparently in red and blue, respectively.
(Right) All-atom explicit-solvent simulation of the ProTα–protamine condensate (total time 1 μs). One ProTα chain is highlighted in red (chain 30), and six interacting protamine chains are shown in three different shades of purple. Other surrounding ProTα and protamine chains are shown semi-transparently in red and purple, respectively.

Both videos are centered on the center of mass of the highlighted ProTα chain. The video is shown at 2 ns per frame. To slightly smooth the motion, a filter with a time constant of 4 ns was applied to all frames. Protein hydrogen atoms, water molecules, and ions were omitted for clarity. (YouTube link to high-resolution version:
https://www.youtube.com/watch?v=E4Idah1J3N8&ab_channel=MilosIvanovic)

Video S2 (separate file)

(Left) All-atom explicit-solvent simulation of the ProTα–H1 condensate (total time 50 ns). One ProTα chain is highlighted in red (chain 59), and four interacting H1 chains are shown in different shades of blue. Other surrounding ProTα and H1 chains are shown semi-transparently in red and blue, respectively.

(Right) All-atom explicit-solvent simulation of the ProTα–protamine condensate (total time 50 ns). One ProTα chain is highlighted in red (chain 30), and six interacting protamine chains are shown in three different shades of purple. Other surrounding ProTα and protamine chains are shown semi transparently in red and purple, respectively.

Both videos are centered on the center of mass of the highlighted ProTα chain. The video is shown at 100 ps per frame. To slightly smooth the motion, a filter with a time constant of 200 ps was applied to all frames. Protein hydrogen atoms, water molecules, and ions were omitted for clarity. (YouTube link to high-resolution version:
https://www.youtube.com/watch?v=4G9GOYp-Fmw&ab_channel=MilosIvanovic)

References

1. A. Sottini, A. Borgia, M. B. Borgia, K. Bugge, D. Nettels, A. Chowdhury, P. O. Heidarsson, F. Zosel, R. B. Best, B. B. Kragelund, B. Schuler, Polyelectrolyte interactions enable rapid association and dissociation in high-affinity disordered protein complexes. *Nature Communications* **11**, 5736 (2020).

2. B. Hellenkamp, *et al.*, Precision and accuracy of single-molecule FRET measurements—a multi-laboratory benchmark study. *Nat Methods* **15**, 669–676 (2018).

3. B. Schuler, "Application of Single Molecule Förster Resonance Energy Transfer to Protein Folding" in *Protein Folding Protocols*, Y. Bai, R. Nussinov, Eds. (Humana Press, 2006), pp. 115–138.

4. B. Schuler, A. Soranno, H. Hofmann, D. Nettels, Single-Molecule FRET Spectroscopy and the Polymer Physics of Unfolded and Intrinsically Disordered Proteins. *Annual Review of Biophysics* **45**, 207–231 (2016).

5. B. W. V. D. Meer, G. III Coker, S. Y. S. Chen, *Resonance Energy Transfer: Theory and Data* (Vch Pub, 1994).

6. D. Klose, A. Holla, C. Gmeiner, D. Nettels, I. Ritsch, N. Bross, M. Yulikov, F. H.-T. Allain, B. Schuler, G. Jeschke, Resolving distance variations by single-molecule FRET and EPR spectroscopy using rotamer libraries. *Biophysical Journal* **120**, 4842–4858 (2021).

7. H. Zhao, P. H. Brown, P. Schuck, On the Distribution of Protein Refractive Index Increments. *Biophysical Journal* **100**, 2309–2317 (2011).

8. R. Barer, S. Tkaczyk, Refractive Index of Concentrated Protein Solutions. *Nature* **173**, 821–822 (1954).

9. W. Zheng, G. H. Zerze, A. Borgia, J. Mittal, B. Schuler, R. B. Best, Inferring properties of disordered chains from FRET transfer efficiencies. *J. Chem. Phys.* **148**, 123329 (2018).

10. M. Aznauryan, L. Delgado, A. Soranno, D. Nettels, J. Huang, A. M. Labhardt, S. Grzesiek, B. Schuler, Comprehensive structural and dynamical view of an unfolded protein from the combination of single-molecule FRET, NMR, and SAXS. *Proceedings of the National Academy of Sciences* **113**, E5389–E5398 (2016).

11. A. Borgia, M. B. Borgia, K. Bugge, V. M. Kissling, P. O. Heidarsson, C. B. Fernandes, A. Sottini, A. Soranno, K. J. Buholzer, D. Nettels, B. B. Kragelund, R. B. Best, B. Schuler, Extreme disorder in an ultrahigh-affinity protein complex. *Nature* **555**, 61–66 (2018).

12. N. Galvanetto, M. T. Ivanović, A. Chowdhury, A. Sottini, M. F. Nüesch, D. Nettels, R. B. Best, B. Schuler, Extreme dynamics in a biomolecular condensate. *Nature* **619**, 876–883 (2023).

13. E. W. Martin, A. S. Holehouse, I. Peran, M. Farag, J. J. Incicco, A. Bremer, C. R. Grace, A. Soranno, R. V. Pappu, T. Mittag, Valence and patterning of aromatic residues determine the phase behavior of prion-like domains. *Science* **367**, 694–699 (2020).

14. T. Dertinger, V. Pacheco, I. von der Hocht, R. Hartmann, I. Gregor, J. Enderlein, Two-Focus Fluorescence Correlation Spectroscopy: A New Tool for Accurate and Absolute Diffusion Measurements. *ChemPhysChem* **8**, 433–443 (2007).

15. J.-Y. Tinevez, N. Perry, J. Schindelin, G. M. Hoopes, G. D. Reynolds, E. Laplantine, S. Y. Bednarek, S. L. Shorte, K. W. Eliceiri, TrackMate: An open and extensible platform for single-particle tracking. *Methods* **115**, 80–90 (2017).

16. T.-H. Fan, J. K. G. Dhont, R. Tuinier, Motion of a sphere through a polymer solution. *Phys. Rev. E* **75**, 011803 (2007).

17. R. Tuinier, J. K. G. Dhont, T.-H. Fan, How depletion affects sphere motion through solutions containing macromolecules. *EPL* **75**, 929 (2006).

18. L.-H. Cai, S. Panyukov, M. Rubinstein, Mobility of Nonsticky Nanoparticles in Polymer Liquids. *Macromolecules* **44**, 7853–7863 (2011).

19. M. Rubinstein, R. H. Colby, *Polymer Physics* (OUP Oxford, 2003).

20. M. Muthukumar, *Physics of Charged Macromolecules: Synthetic and Biological Systems* (Cambridge University Press, 2023).

21. H. Hofmann, A. Soranno, A. Borgia, K. Gast, D. Nettels, B. Schuler, Polymer scaling laws of unfolded and intrinsically disordered proteins quantified with single-molecule spectroscopy. *PNAS* **109**, 16155–16160 (2012).

22. L. J. Fetters, D. J. Lohse, D. Richter, T. A. Witten, A. Zirkel, Connection between Polymer Molecular Weight, Density, Chain Dimensions, and Melt Viscoelastic Properties. *Macromolecules* **27**, 4639–4647 (1994).

23. R. S. Hoy, M. Kröger, Unified Analytic Expressions for the Entanglement Length, Tube Diameter, and Plateau Modulus of Polymer Melts. *Phys. Rev. Lett.* **124**, 147801 (2020).

24. A. Soranno, B. Buchli, D. Nettels, R. R. Cheng, S. Müller-Späth, S. H. Pfeil, A. Hoffmann, E. A. Lipman, D. E. Makarov, B. Schuler, Quantifying internal friction in unfolded and intrinsically disordered proteins with single-molecule spectroscopy. *PNAS* **109**, 17800–17806 (2012).

25. I. V. Gopich, D. Nettels, B. Schuler, A. Szabo, Protein dynamics from single-molecule fluorescence intensity correlation functions. *The Journal of Chemical Physics* **131**, 095102 (2009).

26. D. Nettels, I. V. Gopich, A. Hoffmann, B. Schuler, Ultrafast dynamics of protein collapse from single-molecule photon statistics. *Proceedings of the National Academy of Sciences* **104**, 2655–2660 (2007).

27. P. E. Rouse, A Theory of the Linear Viscoelastic Properties of Dilute Solutions of Coiling Polymers. *The Journal of Chemical Physics* **21**, 1272–1280 (1953).

28. B. H. Zimm, Dynamics of Polymer Molecules in Dilute Solution: Viscoelasticity, Flow Birefringence and Dielectric Loss. *The Journal of Chemical Physics* **24**, 269–278 (1956).

29. P. G. De Gennes, Dynamics of Entangled Polymer Solutions. I. The Rouse Model. *Macromolecules* **9**, 587–593 (1976).

30. P. G. de Gennes, Reptation of a Polymer Chain in the Presence of Fixed Obstacles. *The Journal of Chemical Physics* **55**, 572–579 (1971).

31. M. Doi, S. F. Edwards, *The Theory of Polymer Dynamics* (Clarendon Press, 1994).

32. D. E. Makarov, Spatiotemporal correlations in denatured proteins: The dependence of fluorescence resonance energy transfer (FRET)-derived protein reconfiguration times on the location of the FRET probes. *The Journal of Chemical Physics* **132**, 035104 (2010).

33. V. A. Harmandaris, V. G. Mavrantzas, D. N. Theodorou, M. Kröger, J. Ramírez, H. C. Öttinger, D. Vlassopoulos, Crossover from the Rouse to the Entangled Polymer Melt Regime: Signals from Long, Detailed Atomistic Molecular Dynamics Simulations, Supported by Rheological Experiments. *Macromolecules* **36**, 1376–1387 (2003).

34. R. Everaers, Rheology and Microscopic Topology of Entangled Polymeric Liquids. *Science* **303**, 823–826 (2004).

35. R. B. Best, W. Zheng, J. Mittal, Balanced Protein–Water Interactions Improve Properties of Disordered Proteins and Non-Specific Protein Association. *J. Chem. Theory Comput.* **10**, 5113–5124 (2014).

36. K. Lindorff-Larsen, S. Piana, K. Palmo, P. Maragakis, J. L. Klepeis, R. O. Dror, D. E. Shaw, Improved side-chain torsion potentials for the Amber ff99SB protein force field. *Proteins: Structure, Function, and Bioinformatics* **78**, 1950–1958 (2010).

37. J. L. F. Abascal, C. Vega, A general purpose model for the condensed phases of water: TIP4P/2005. *The Journal of Chemical Physics* **123**, 234505 (2005).

38. Y. Luo, B. Roux, Simulation of Osmotic Pressure in Concentrated Aqueous Salt Solutions. *J. Phys. Chem. Lett.* **1**, 183–189 (2010).

39. G. Bussi, D. Donadio, M. Parrinello, Canonical sampling through velocity rescaling. *The Journal of Chemical Physics* **126**, 014101 (2007).

40. M. Parrinello, A. Rahman, Polymorphic transitions in single crystals: A new molecular dynamics method. *Journal of Applied Physics* **52**, 7182–7190 (1981).

41. T. Darden, D. York, L. Pedersen, Particle mesh Ewald: An N·log(N) method for Ewald sums in large systems. *The Journal of Chemical Physics* **98**, 10089–10092 (1993).

42. B. Hess, H. Bekker, H. J. C. Berendsen, J. G. E. M. Fraaije, LINCS: A linear constraint solver for molecular simulations. *Journal of Computational Chemistry* **18**, 1463–1472 (1997).

43. M. J. Abraham, T. Murtola, R. Schulz, S. Páll, J. C. Smith, B. Hess, E. Lindahl, GROMACS: High performance molecular simulations through multi-level parallelism from laptops to supercomputers. *SoftwareX* **1**, 19–25 (2015).

44. W. Zheng, G. L. Dignon, N. Jovic, X. Xu, R. M. Regy, N. L. Fawzi, Y. C. Kim, R. B. Best, J. Mittal, Molecular Details of Protein Condensates Probed by Microsecond Long Atomistic Simulations. *J. Phys. Chem. B* **124**, 11671–11679 (2020).

45. J. Karanicolas, C. L. Brooks III, The origins of asymmetry in the folding transition states of protein L and protein G. *Protein Science* **11**, 2351–2361 (2002).

46. P. Rotkiewicz, J. Skolnick, Fast procedure for reconstruction of full-atom protein models from reduced representations. *Journal of Computational Chemistry* **29**, 1460–1465 (2008).

47. A. Vitalis, R. V. Pappu, ABSINTH: A new continuum solvation model for simulations of polypeptides in aqueous solutions. *Journal of Computational Chemistry* **30**, 673–699 (2009).

48. G. Hummer, Position-dependent diffusion coefficients and free energies from Bayesian analysis of equilibrium and replica molecular dynamics simulations. *New J. Phys.* **7**, 34 (2005).

49. R. B. Best, G. Hummer, Coordinate-dependent diffusion in protein folding. *Proceedings of the National Academy of Sciences* **107**, 1088–1093 (2010).

50. D. J. Bicout, A. Szabo, Electron transfer reaction dynamics in non-Debye solvents. *The Journal of Chemical Physics* **109**, 2325–2338 (1998).

51. W. H. Press, Ed., *Numerical recipes: the art of scientific computing*, 3rd ed (Cambridge University Press, 2007).

52. N.-V. Buchete, G. Hummer, Coarse Master Equations for Peptide Folding Dynamics. *J. Phys. Chem. B* **112**, 6057–6069 (2008).

53. G. H. Zerze, J. Mittal, R. B. Best, Diffusive Dynamics of Contact Formation in Disordered Polypeptides. *Phys. Rev. Lett.* **116**, 068102 (2016).

54. R. B. Best, G. Hummer, W. A. Eaton, Native contacts determine protein folding mechanisms in atomistic simulations. *Proceedings of the National Academy of Sciences* **110**, 17874–17879 (2013).

55. S. Kim, J. Huang, Y. Lee, S. Dutta, H. Y. Yoo, Y. M. Jung, Y. Jho, H. Zeng, D. S. Hwang, Complexation and coacervation of like-charged polyelectrolytes inspired by mussels. *Proceedings of the National Academy of Sciences* **113**, E847–E853 (2016).

56. Y. Hong, S. Najafi, T. Casey, J.-E. Shea, S.-I. Han, D. S. Hwang, Hydrophobicity of arginine leads to reentrant liquid-liquid phase separation behaviors of arginine-rich proteins. *Nat Commun* **13**, 7326 (2022).

57. Y. An, M. A. Webb, W. M. Jacobs, Active learning of the thermodynamics-dynamics trade-off in protein condensates. *Science Advances* **10**, eadj2448 (2024).

58. L. Li, S. Srivastava, M. Andreev, A. B. Marciel, J. J. de Pablo, M. V. Tirrell, Phase Behavior and Salt Partitioning in Polyelectrolyte Complex Coacervates. *Macromolecules* **51**, 2988–2995 (2018).

59. S. Qamar, G. Wang, S. J. Randle, F. S. Ruggeri, J. A. Varela, J. Q. Lin, E. C. Phillips, A. Miyashita, D. Williams, F. Ströhl, W. Meadows, R. Ferry, V. J. Dardov, G. G. Tartaglia, L. A. Farrer, G. S. K. Schierle, C. F. Kaminski, C. E. Holt, P. E. Fraser, G. Schmitt-Ulms, D. Klenerman, T. Knowles, M. Vendruscolo, P. S. George-Hyslop, FUS Phase Separation Is Modulated by a Molecular Chaperone and Methylation of Arginine Cation-π Interactions. *Cell* **173**, 720-734.e15 (2018).

60. M. Paloni, G. Bussi, A. Barducci, Arginine multivalency stabilizes protein/RNA condensates. *Protein Science* **30**, 1418–1426 (2021).

61. J. Wang, J.-M. Choi, A. S. Holehouse, H. O. Lee, X. Zhang, M. Jahnel, S. Maharana, R. Lemaitre, A. Pozniakovsky, D. Drechsel, I. Poser, R. V. Pappu, S. Alberti, A. A. Hyman, A Molecular Grammar Governing the Driving Forces for Phase Separation of Prion-like RNA Binding Proteins. *Cell* **174**, 688-699.e16 (2018).

62. R. M. Vernon, P. A. Chong, B. Tsang, T. H. Kim, A. Bah, P. Farber, H. Lin, J. D. Forman-Kay, Pi-Pi contacts are an overlooked protein feature relevant to phase separation. *eLife* **7**, e31486 (2018).

63. M. Muthukumar, Dynamics of polyelectrolyte solutions. *The Journal of Chemical Physics* **107**, 2619–2635 (1997).

64. M.-T. Wei, S. Elbaum-Garfinkle, A. S. Holehouse, C. C.-H. Chen, M. Feric, C. B. Arnold, R. D. Priestley, R. V. Pappu, C. P. Brangwynne, Phase behaviour of disordered proteins underlying low density and high permeability of liquid organelles. *Nature Chemistry* **9**, 1118–1125 (2017).

65. I. Alshareedah, W. M. Borcherds, S. R. Cohen, A. Singh, A. E. Posey, M. Farag, A. Bremer, G. W. Strout, D. T. Tomares, R. V. Pappu, T. Mittag, P. R. Banerjee, Sequence-specific interactions determine viscoelasticity and ageing dynamics of protein condensates. *Nat. Phys.* 1–10 (2024). https://doi.org/10.1038/s41567-024-02558-1.

66. C. Ji, E. Girardi, N. Fawzi, J. Tang, Density and viscosity measurements on the liquid condensates of FUS protein low-complexity domain. *APS March Meeting* **64**, K65.00005 (2019).

67. A. C. Murthy, G. L. Dignon, Y. Kan, G. H. Zerze, S. H. Parekh, J. Mittal, N. L. Fawzi, Molecular interactions underlying liquid−liquid phase separation of the FUS low-complexity domain. *Nat Struct Mol Biol* **26**, 637–648 (2019).

68. M. Staropoli, M. Kruteva, J. Allgaier, A. Wischnewski, W. Pyckhout-Hintzen, Supramolecular Dimerization in a Polymer Melt from Small-Angle X-ray Scattering and Rheology: A Miscible Model System. *Polymers* **12**, 880 (2020).

69. I. Alshareedah, T. Kaur, P. R. Banerjee, “Chapter Six - Methods for characterizing the material properties of biomolecular condensates” in *Methods in Enzymology*, Liquid-Liquid Phase Coexistence and Membraneless Organelles., C. D. Keating, Ed. (Academic Press, 2021), pp. 143–183.
